# Non-ureolytic EICP as a novel enzymatic pathway for sustainable soil stabilization

**DOI:** 10.1038/s41598-025-13525-y

**Published:** 2025-08-01

**Authors:** Sajjad Deylaghian, Ehsan Nikooee, Aniseh Seyedi, Ali Niazi, Thomas Nagel

**Affiliations:** 1 Department of Civil and Environmental Engineering, Shiraz University, Shiraz, Iran; 2 Department of Civil Engineering, Shahid Bahonar University, Kerman, Iran; 3 Institute of Biotechnology, Shiraz University, Shiraz, Iran; 4 Geotechnical Institute, TU Bergakademie Freiberg, Freiberg, Germany; 5 Department of Environmental Informatics, Helmholtz Centre for Environmental Research GmbH (UFZ), Leipzig, Germany; 6 Freiberg Center for Water Research (ZeWaF), TU Bergakademie Freiberg, Freiberg, Germany

**Keywords:** Formate dehydrogenase (FDH), Non-ureolytic EICP, Sustainable ground improvement, Cleaner EICP, Ammonium-free EICP, Civil engineering, Biogeochemistry

## Abstract

In order to address the challenges of the modern era, such as population growth and greenhouse gas emissions, sustainable soil improvement techniques have been in high demand. In a quest to find more sustainable ground improvement methods, microbially induced carbonate precipitation (MICP) and enzyme-induced carbonate precipitation (EICP) have attracted researchers worldwide. For the first time, this study investigates a non-ureolytic enzyme-induced carbonate precipitation method using formate dehydrogenase (FDH) as a sustainable approach for soil stabilization. Unlike traditional EICP and MICP methods, this technique avoids ammonium by-products, providing an environmentally friendly alternative. FDH activity was optimized at pH 7.6 and 37 °C. Its thermal stability was found to be reasonably suitable for biogeotechnical applications, maintaining over 90% efficiency up to 50 °C and achieving over 50% efficiency at 60 °C. The compressive strength of non-ureolytic EICP-treated soil samples and calcium carbonate content were investigated across various treatment cycles on poorly graded sand (SP). The optimal enzyme concentration was 75 mg/L with 50 g/L of calcium formate. Soil treated with this method exhibited a 31-fold increase in strength after five treatment cycles, with 1.89% calcium carbonate precipitation. Microstructural analyses (XRD, FTIR, SEM) and calcium carbonate content measurements using the Bernard calcimeter confirmed the formation of carbonate crystals between soil grains, contributing to this strength enhancement.

**Supplementary Information:**

The online version contains supplementary material available at 10.1038/s41598-025-13525-y.

## Introduction

Soil stabilization remains perilously reliant on carbon-intensive additives like cement, a material responsible for nearly 8% of global CO₂ emissions^1,2^. As climate change has increasingly dire consequences, new soil stabilization techniques and paradigms must be investigated. Decoupling stabilization from its carbon legacy demands the urgent adoption of low-emission alternatives that reconcile mechanical performance with sustainability goals. The construction sector’s capacity to pivot toward such innovations will indelibly shape whether our future built environments evolve as climate adversaries or climate-benign assets.

Recently, various bio-inspired and bio-mediated soil stabilization technologies have been developed as potential alternatives to conventional high-carbon-footprint treatments^3–8^. Microbially-induced calcium carbonate precipitation (MICP) is a growing bio-inspired technology that utilizes urease-producing microorganisms to hydrolyze urea and precipitate calcium carbonate (CaCO₃) for soil stabilization^9–17^. However, challenges associated with exocultivation, transport of bacterial solutions^18,19^and nutrient-dependent bacterial activity are some of the problems that can affect the viability and efficiency of MICP when trying to achieve consistency and reliable soil strengthening. Moreover, the bacteria should be smaller than the soil pore throats. Otherwise, their transport is limited, hindering biocementation across all pore sizes^20–22^. Cell-free enzymes have recently been proposed as an alternative to microorganism-based ground improvement techniques to overcome these challenges. In enzyme-induced carbonate precipitation (EICP), enzyme-catalyzing reactions leading to cementation are directly employed, alleviating the need for living cells and the limitations discussed earlier^23–25^.

Nemati and Voordouw were the first to utilize the urease enzyme extracted from plant sources instead of bacteria to reduce soil permeability^26^. Afterward, Bang et al. employed the urease enzyme for dust suppression^27^. In the following, Yasuhara et al. utilized the urease enzyme to enhance the rate and volume of calcium carbonate precipitation during the grouting and cementing of sand^28,29^. In another study, Majumdar et al. investigated the application of a novel bacterial protein, or crude enzyme from *Thermoanaerobactor Fermicutes*, in pozzolana cement-based mortar specimens^29^. The crude proteins were extracted by centrifugation and then lyophilized from the supernatants. The samples’ strength and durability were enhanced by this protein, which also improved their ability to heal cracks^30^. In another study, Kavazanjian and Hamdan explored urease’s potential for geotechnical applications^31^. A critical benefit of the enzyme is its small size and water solubility, enabling it to pass through the pores of soils with smaller grains, such as silts. MICP is, instead, generally effective in strengthening fine to medium-sized sand or larger-grain soils, with reduced performance in finer-grained soils^22,31–33^.

During recent decades, extensive research has been conducted on the EICP for various applications, including erosion and dust control^34–37^construction materials^38,39^as well as reducing the swelling potential of expansive soils^23^and many other geotechnical and geo-environmental applications^40,41^. However, its practical implementation encounters several challenges, including limited efficiency at high temperatures, uneven solidification, decreased performance under pressure, and limited long-term applicability in field conditions. Recent studies have suggested various modifications to overcome these limitations and enhance the robustness of the EICP process^42–44^. Existing studies on EICP have primarily focused on carbonate precipitation facilitated by the urease enzyme^25,45–48^. Urease catalyzes the hydrolysis of urea into ammonium (NH₄⁺) and carbonate (CO₃⁻^2^ ions. In the presence of calcium ions (Ca²⁺), calcium carbonate (CaCO₃) precipitates, as shown in Eqs. (1) and (2):

1 $$\:{\text{C}\text{O}\left({\text{N}\text{H}}_{2}\right)}_{2}+2{\text{H}}_{2}\text{O}\:\underrightarrow{\:\text{U}\text{r}\text{e}\text{a}\text{s}\text{e}\:}\:\:{{2\text{N}\text{H}}_{4}}^{+}+\:{{\text{C}\text{O}}_{3}}^{2-}$$

2 $$\:{\:\:\text{C}\text{a}}^{2+}+{{\text{C}\text{O}}_{3}}^{2-}\rightarrow{\text{C}\text{a}\text{C}\text{O}}_{3}\downarrow$$

The carbonate precipitation binds soil particles together, enhancing soil strength and stability. The urease enzyme can be extracted from diverse biological sources, namely, algae, fungi, and bacteria such as *Sporosarcina Pasteurii*^49^. The first documented extraction of urease from jack beans was reported by Sumner et al. in 1934^50^. Due to its rapid and controllable reaction, EICP has emerged as a versatile and efficient method for soil improvement. However, the hydrolysis of urea produces ammonium (NH₄⁺) as a byproduct. It is notable that high ammonium level raises environmental concerns. This process increases soil nitrogen levels and alters the soil pH in an undesirable manner^51^. Extreme soil salinity and groundwater contamination can occur if ammonium is not properly handled^52–54^.

Moreover, the excess of ammonium exceeds the demands for watering and pruning plants, potentially leading to overgrowth in the available space. Overgrowth of the plants leads to their premature death. The ammonium byproduct can also contribute to water pollution, thereby harming aquatic organisms. Thus, appropriate management and measures should be established to control ammonium waste using urease for EICP and MICP^55^. A different microorganism or enzyme with alternative metabolic pathways could address these environmental issues.

Hemayati et al. utilized heterotrophic bacteria and an alternative metabolic pathway to induce carbonate precipitation through MICP^56^. This method did not result in the production of ammonium ions. They utilized calcium formate as a carbon source to produce energy for the growth of microorganisms. While their selected metabolic pathway was ammonium-free and a greener pathway for soil stabilization, their proposed method was tethered to bacterial cells to produce the formate dehydrogenase (FDH) enzyme required for formate decomposition^24,56^. The FDH enzyme produced by bacteria oxidizes formate and produces carbonate ions required to form calcium carbonate.

The transportation and cultivation of bacterial cells for MICP soil stabilization are faced with several challenges. In contrast, enzymes benefit from smaller (molecular) sizes (compared to bacteria), allowing more homogenous and efficient soil stabilization. In the current study, the feasibility of using FDH enzyme directly in formate-driven EICP as an alternative to formate-driven MICP has been investigated. This novel ammonium-free enzymatic approach provides a green alternative to conventional urease-based EICP for soil stabilization, eliminating the environmental concerns associated with producing ammonium ions in the conventional EICP.

Formate dehydrogenase (FDH) is an enzyme that yields energy-generating processes during anaerobic metabolism in various organisms. This enzyme is essential in various metabolic pathways where formate (-HCOO-, a simple one-carbon molecule) is an energy source or carbon donor. The catalytic activity of FDH represents a necessary process in which formate is oxidized to carbon dioxide. At the same time, NAD^+^ (Nicotinamide adenine dinucleotide, a reducing agent) is reduced to NADH in this metabolic pathway. The FDH enables the recycling of hydrogen and carbon dioxide, which are subsequently integrated into carbon metabolism, allowing formate to function as a carbon source. FDH also aids in detoxifying formate and maintaining cellular homeostasis, supporting the creation of redox equivalents in the conversion of NADH, which it facilitates as a cofactor of numerous metabolic reactions.

In formate-driven Enzyme-Induced Carbonate Precipitation (EICP) systems, the process initiates with the dissociation of calcium formate (Ca (HCOO)₂) in water, releasing calcium (Ca²⁺) and formate (HCOO⁻) ions. The formate ions are then oxidized to carbon dioxide (CO₂) by formate dehydrogenase (FDH), an enzyme that facilitates this specific reaction. The CO₂ produced can dissolve in water, forming carbonic acid (H₂CO₃), which subsequently dissociates into bicarbonate (HCO₃⁻) and protons (H⁺), potentially lowering the pH of the environment. The presence of calcium ions facilitates the precipitation of calcium carbonate (CaCO₃), thereby enhancing soil stability through the process of particle cementation. The reactions taking place within the nonureolytic enzymatic pathway of carbonate precipitation are outlined in the following Eqs. (3–7):

3 $$\:{\text{C}\text{a}\left(\text{H}\text{C}\text{O}\text{O}\right)}_{2}\rightarrow{\text{C}\text{a}}^{2+}+\:{2\text{H}\text{C}\text{O}\text{O}}^{-}$$

4 $$\:{\text{H}\text{C}\text{O}\text{O}}^{-}+{\text{N}\text{A}\text{D}}^{+}\underrightarrow{\text{F}\text{D}\text{H}}\:\:{\text{C}\text{O}}_{2}+\text{N}\text{A}\text{D}\text{H}+{\text{H}}^{+}$$

5 $$\:{\text{C}\text{O}}_{2}+{\text{H}}_{2}\text{O}\leftrightarrow{\text{H}}_{2}{\text{C}\text{O}}_{3}$$

6 $$\:{\text{H}}_{2}{\text{C}\text{O}}_{3}\leftrightarrow{\text{H}{\text{C}\text{O}}_{3}}^{-}+{\text{H}}^{+}$$

7 $$\:{\text{C}\text{a}}^{2+}+{\text{H}{\text{C}\text{O}}_{3}}^{-}\leftrightarrow{\text{C}\text{a}\text{C}\text{O}}_{3}\downarrow\:+{\text{H}}^{+}$$

This study investigated the efficiency of this alternative EICP pathway under various environmental conditions. The enzyme used in this study was formate dehydrogenase (FDH) from *Candida boidinii*. The stability of the enzyme under environmental conditions was also assessed. The optimal environmental conditions and required compositions for FDH’s best efficiency were investigated, too. Additionally, the extent of carbonate precipitation and the types and morphologies of the resulting crystals were analyzed using scanning electron microscopy (SEM) imaging, X-ray diffraction (XRD), and Fourier Transform Infrared Spectroscopy (FTIR). This urease-free approach to EICP offers an alternative pathway for carbonate precipitation, with the potential to mitigate environmental impacts associated with ammonium byproducts generated in traditional urea-based EICP processes. This alternative pathway minimizes environmental concerns and offers a more sustainable solution for bio-cementation and ground improvement applications.

## Materials and methods

### Soil samples

In this study, aeolian sand (wind-blown sand), which is commonly found in arid regions with a loose, non-cohesive structure and relatively uniform grain size, was utilized. The soil was collected from an area in Iran (coordinates N 30°06’21.4” and E 57°11’33.8”). The soil was classified as problematic due to its gradation, low bearing capacity, and low cohesion. Wind-blown sand, loess, or alluvial deposits are collapsible soils usually deposited in arid or semi-arid environments. These characteristics make this soil a suitable candidate for soil stabilization-type studies. Aeolian sand is a silica sand consisting of quartz (SiO₂).

**Fig. 1:**
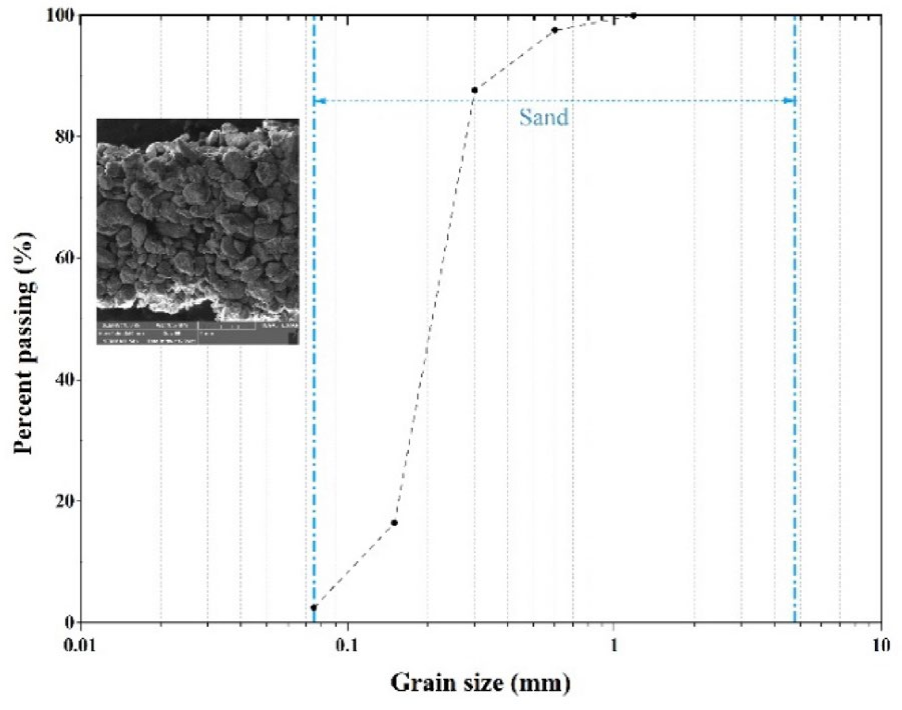
Particle-size distribution of soil used in this study.

**Fig. 2:**
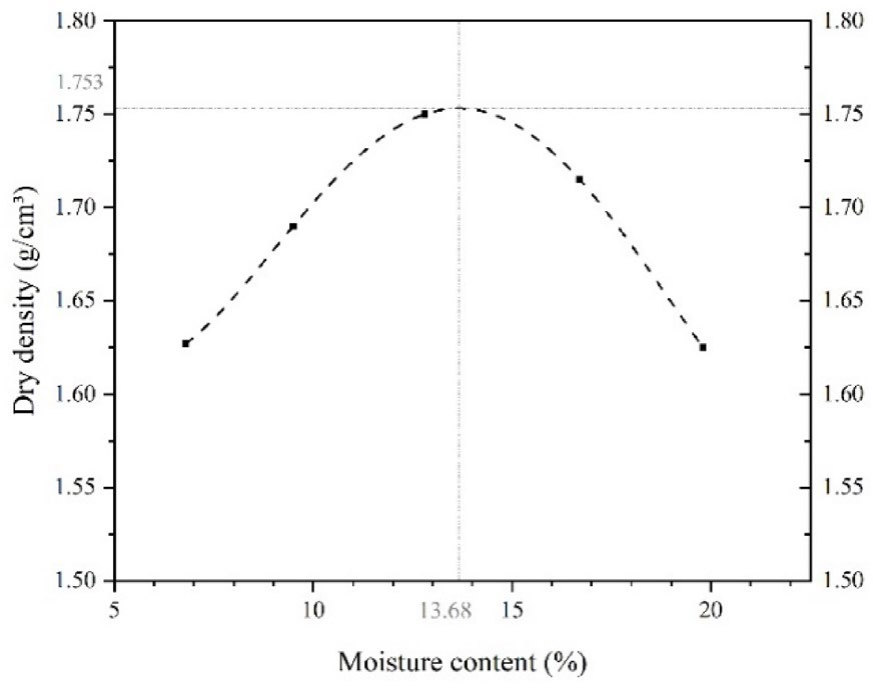
Standard Proctor compaction test curve (Moisture-density relationship curve) of soil used in this study.

A sieve analysis was conducted following ASTM D7928-17 to classify the soil properties. The soil was characterized as poorly graded sand (SP) according to the Unified Soil Classification System (USCS). Its particle size distribution curve is presented in Fig. 1. Following ASTM-D698, a standard compaction test was conducted that provided optimum water content values of 13.68% and a maximum dry density of 1.753 kg/cm³ (see Fig. 2). Engineering and physical soil characteristics were determined following ASTM standards. These characteristics are summarized in Table 1.

**Table 1:** Characteristics of soil samples used in this study.

Engineering properties and physical characteristics (unit)	Standard	Values
Specific gravity of the grains (g/cm^3^)	ASTM C127-88	2.60
in-situ density (g/cm^3^)	ASTM D 1556	1.33
D_10_ (µm)	ASTM D 2487	130
D_30_ (µm)	ASTM D 2487	180
D_60_ (µm)	ASTM D 2487	230
Maximum dry density (g/cm^3^)	ASTM D698	1.753
Optimum moisture content (%)	ASTM D698	13.68
Uniformity coefficient, C_U_	ASTM D6913	1.08
Curvature coefficient, C_C_	ASTM D6913	1.77
Unified soil classification system (USCS)	ASTM D2487	SP

### Chemical materials

Calcium formate was used as the calcium source due to its ability to produce pure calcium carbonate with only CO₂ and H₂O as by-products, eliminating ammonia formation. It also serves as both a calcium and a carbonate source. Potassium phosphate was used as a buffering agent, and NAD⁺ acted as the redox cofactor for the enzymatic reaction. All chemicals were analytical grade and supplied by Sigma Aldrich Merck (Germany).

### Formate dehydrogenase (FDH)

This study used Formate Dehydrogenase (FD) from *Candida boidinii*, supplied by Creative Enzyme. The enzyme was in liquid form with a molecular weight of approximately 41 kDa. FDH is classified as formate NAD⁺ oxidoreductase (EC 1.2.1.2), which catalyzes formate oxidation to carbon dioxide (CO₂) and simultaneously reduces NAD⁺ to NADH. The enzyme exhibits an activity of approximately 1 unit per milligram of protein. The activity was related to the standard unit definition, where one unit (U) is defined as the amount of enzyme required to convert one µmole of formic acid to NADH and CO_2_ per minute in the presence of NAD⁺ in a potassium phosphate buffer at pH 7.6 and 25 °C. The enzyme was stored at 4 °C before and during experimental procedures. The FDH was tested in the laboratory under standard conditions (25 °C, pH 7.6) to ensure consistency and accuracy in its activity.

The FDH enzymatic pathway is essential for energy production in various organisms, playing a critical role in cellular redox balance, energy production, and carbon metabolism. FDH has numerous industrial applications, including biocatalysis, carbon capture, and NADH regeneration. This application has fostered its applications in environmental engineering^57–60^. Hence, the direct application of cell-free enzymes in applications such as soil stabilization is deemed necessary and has been examined in this study.

### NAD^+^

Nicotinamide adenine dinucleotide (NAD) is a cofactor that contributes to the metabolism of all living cells. It exists in both oxidized (NAD^+^) and reduced (NADH) forms. It functions as a redox coenzyme, paired with NADH, and serves as a substrate for NAD-dependent enzymes, such as FDH. As a coenzyme, NAD⁺ is necessary in redox reactions by acting as a hydrogen and electron acceptor. In the present study, NAD⁺ was utilized as a cofactor for FDH to enable the enzymatic oxidation of formate, a key step in the proposed formate-driven EICP system^61,62^.

Candida boidinii formate dehydrogenase (CbFDH) demonstrates a high affinity for the NAD⁺ cofactor. Consequently, the enzyme operates efficiently even at low NAD⁺ concentrations. This makes CbFDH highly suitable for biocatalytic applications and enzymatic CO₂ conversionwhere minimizing cofactor costs is important^63^.

## Experimental program

### Optimization of environmental conditions and compositions for FDH

#### FDH activity and optimization of environmental conditions

FDH activity is affected by environmental conditions such as temperature and pH. Bacterial FDH exhibits an optimum range of temperature between 30 °C and 37 °C, whereas plant FDH shows a lower optimum range. High temperatures can denature the enzyme, while low temperatures reduce catalytic efficiency. Most FDH enzymes show optimal activity between pH 7.0 and 7.7 ^64–66^.

In this study, the activity of FDH was determined at various temperatures and pH levels using spectrophotometry at 340 nm. The formation of NADH is indicated by absorption at 340 nm. The reaction mixture consists of 41 mM potassium phosphate buffer (pH 7.6), calcium formate as the substrate at 50 g/L, NAD⁺ as the cofactor at 290 g/L, and FDH enzyme at 75 mg/L. The solution was first incubated at 25 °C for 20 min. The solution’s optical density at 340 nm was measured. Then, the enzyme activity was calculated using Beer-Lambert’s Law^67,68^. In this method, the molar extinction coefficient of NADH at 340 nm was used as an indicator of FDH activity. The activity was related to the standard unit definition, where one unit (U) is defined as the amount of enzyme required to convert one µmole of formic acid to NADH and CO₂ per minute in the presence of NAD⁺ in a potassium phosphate buffer at pH 7.6 and 25 °C.

**Fig. 3:**
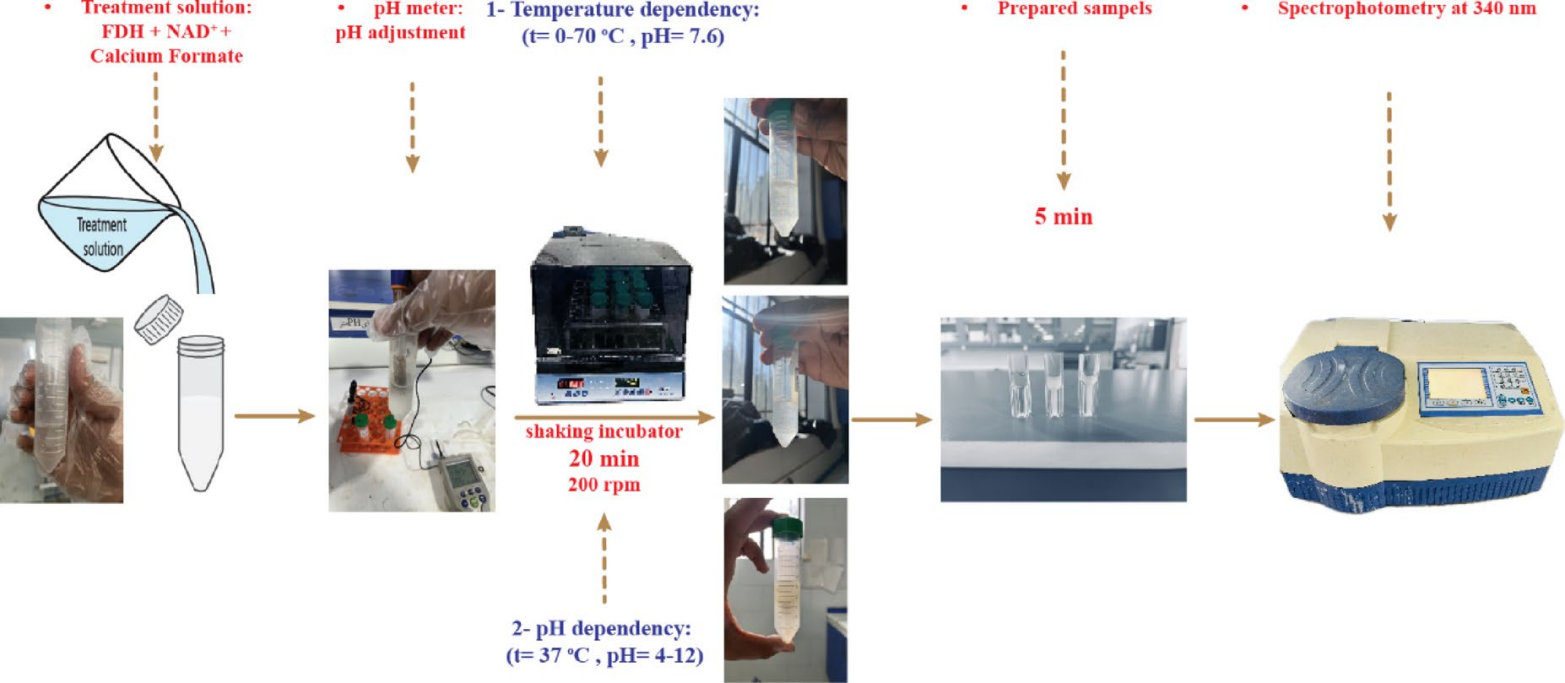
Presentation of the procedure for tests optimizing environmental conditions.

To investigate the impact of temperature on enzyme activity, the solution was initially incubated at different temperatures for 20 minutes^67^. The absorbance change was measured at 340 nm to determine enzyme activity. The enzyme activity was then calculated as stated. To investigate the pH dependency of the FDH enzyme, the buffer pH was varied while maintaining a constant temperature of 37 °C, and the enzyme activity was recorded. The testing procedure to optimize environmental conditions is illustrated in Fig. 3.

The relative activity refers to the activity of an enzyme at a specific temperature, normalized to the activity under standard conditions (pH 7.6 and 25 °C). In this study, the relative activity at different temperatures and pH levels was calculated by comparing the measured activity to that under standard conditions. All results were averages of three individual measurements. The remaining activity is the residual activity of the enzyme after being exposed to or incubated for 20 min at a specified temperature or condition. The activity exhibited by the enzyme after such harsh conditions indicates its resilience against environmental stresses.

#### Optimization of compositions for FDH

**Fig. 4:**
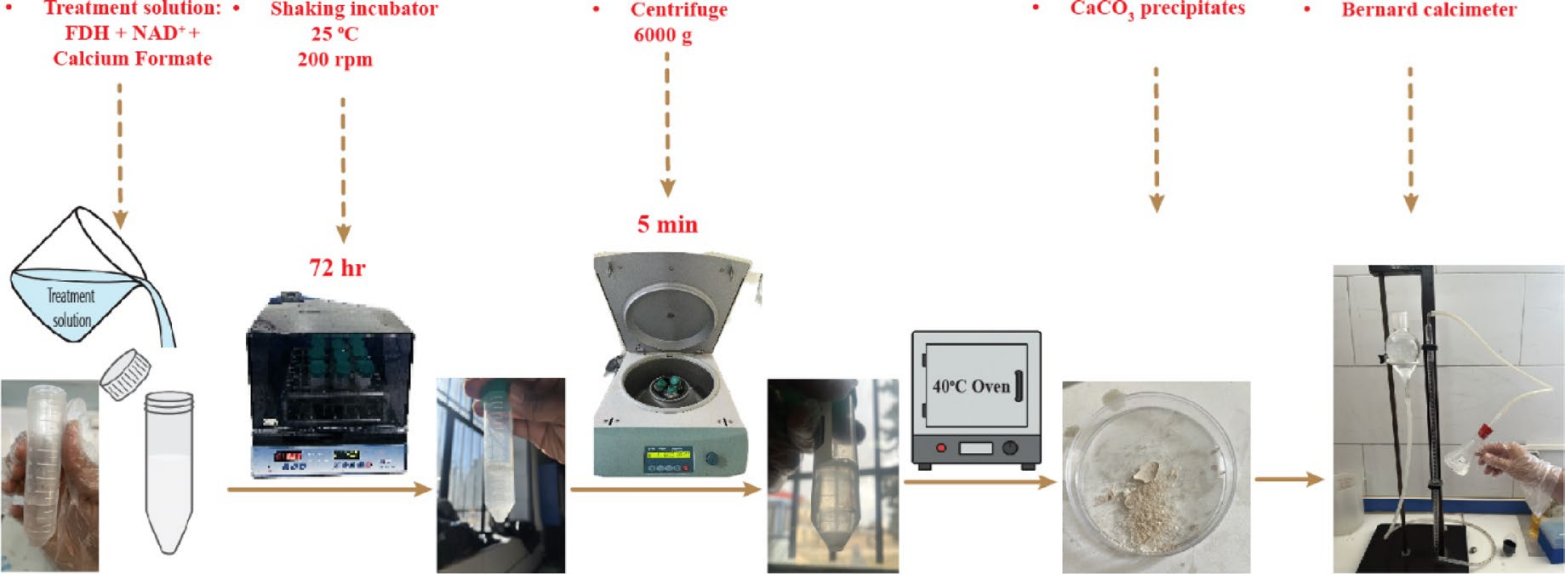
Presentation of the procedure for optimizing precipitated CaCO_3_ amount.

The calcium carbonate content is a governing factor contributing to the efficiency of the soil treatment. Therefore, compositions optimizing the calcium carbonate content can be selected for the next stage, which is testing the mechanical performance of the treated soil samples. In this study, experimental tube samples with enzyme concentrations of 50, 75, and 100 mg/L and calcium formate concentrations of 30 and 50 g/L were prepared to determine the optimal composition of treatment solution ingredients for maximum efficiency. The samples are incubated at the optimal environmental condition (obtained in the previous experimental stage) for 72 h. After incubation, each tube was centrifuged, and the mass of calcium carbonate precipitated in each tube was determined using a Bernard calcimeter^69^. In this experiment, the reaction of CaCO_3_ powder with 1.0 N HCl (ASTM D4373-02) produces CO_2_, and the volume of this gas corresponds to the amount of CaCO_3_. To convert the CO_2_ volume to CaCO_3_ content, a calibration curve was obtained by washing pure CaCO_3_ powder with 1 N HCl and plotting the results against the emitted CO_2_, using a procedure similar to previous studies^56^. The testing procedure for optimizing precipitated CaCO_3_ content is depicted in Fig. 4.

In this study, the conversion rate^70^ (also known as the precipitation ratio^71^ of calcium carbonate precipitation (%) was used to evaluate the efficiency of carbonate formation in both FDH- and urease-based EICP systems. It is defined as the relationship between the theoretical amount of calcium carbonate (CaCO₃) that could precipitate and the actual amount formed under experimental conditions, as presented in Eq. (8). The theoretical maximum was calculated based on the initial molar concentration of the carbon source, formate in the FDH system or urea in the urease system, assuming a 1:1 molar relationship between the carbon source and the resulting CaCO₃.

8 $$\:\text{C}\text{o}\text{n}\text{v}\text{e}\text{r}\text{s}\text{i}\text{o}\text{n}\:\text{r}\text{a}\text{t}\text{e}\:\left(\text{\%}\right)=\left(\frac{\text{m}\text{o}\text{l}\:\text{o}\text{f}\:\text{C}\text{a}\text{C}\text{O}_{3}\:\text{p}\text{r}\text{o}\text{d}\text{u}\text{c}\text{e}\text{d}}{\text{t}\text{h}\text{e}\text{o}\text{r}\text{e}\text{t}\text{i}\text{c}\text{a}\text{l}\:\text{m}\text{o}\text{l}\:\text{o}\text{f}\:\text{C}\text{a}\text{C}\text{O}_{3}\:\text{f}\text{r}\text{o}\text{m}\:\text{t}\text{h}\text{e}\:\text{c}\text{a}\text{r}\text{b}\text{o}\text{n}\:\text{s}\text{o}\text{u}\text{r}\text{c}\text{e}}\:\right)\times\:100\text{\%}$$

### Specimen preparation

Initially, the soil was mixed with the treatment solution at its optimum water content (W_opt_ = 13.86%). The soil mixture was then compacted in three layers within cylindrical molds with a compaction hammer to gain the maximum dry density. The samples were 38 mm in diameter and 76 mm in height. The compacted soil was kept in the molds for at least 72 h at room temperature and humidity for curing. A treatment cycle consisted of treating the sample with the EICP solution and curing it for 72 h. It is worth noting that in the first cycle, the soil was compacted at W_opt_ to achieve the maximum dry density. The EICP solution was increased to one pore volume (~ 27.8 mL) for the subsequent treatment cycles to achieve complete infiltration and uniform distribution of the solution throughout the pore space. This volume is typically greater than W_opt_ and is necessary to fill the entire pore network for effective treatment. Additionally, the bottom and top of the molds were closed with rubber between treatments to minimize solution evaporation, and the samples were stored at room temperature and humidity for 72 h to allow for curing.

After the cycle ended, the residual fluid was drained out from the bottom of the cylinder by puncturing the rubber. The solution was allowed to pass through each specimen. A total of five treatment cycles were performed in this study. Previous studies have also considered treatment cycles in the same range and a similar procedure; therefore, five cycles were selected to ease the comparison of the efficiency of this method with previous studies^40,71–73^. After the final treatment cycle, specimens were rinsed with 28 mL of deionized water. Afterward, they were removed from their molds. All samples were placed in an oven at 40 °C until a constant mass was achieved. Once the oven-drying was complete, specimens were prepared for the UCS Test. After completing the UCS test, the mass of calcium carbonate precipitated in each specimen was determined using the Bernard calcimeter test^69^.

**Fig. 5:**
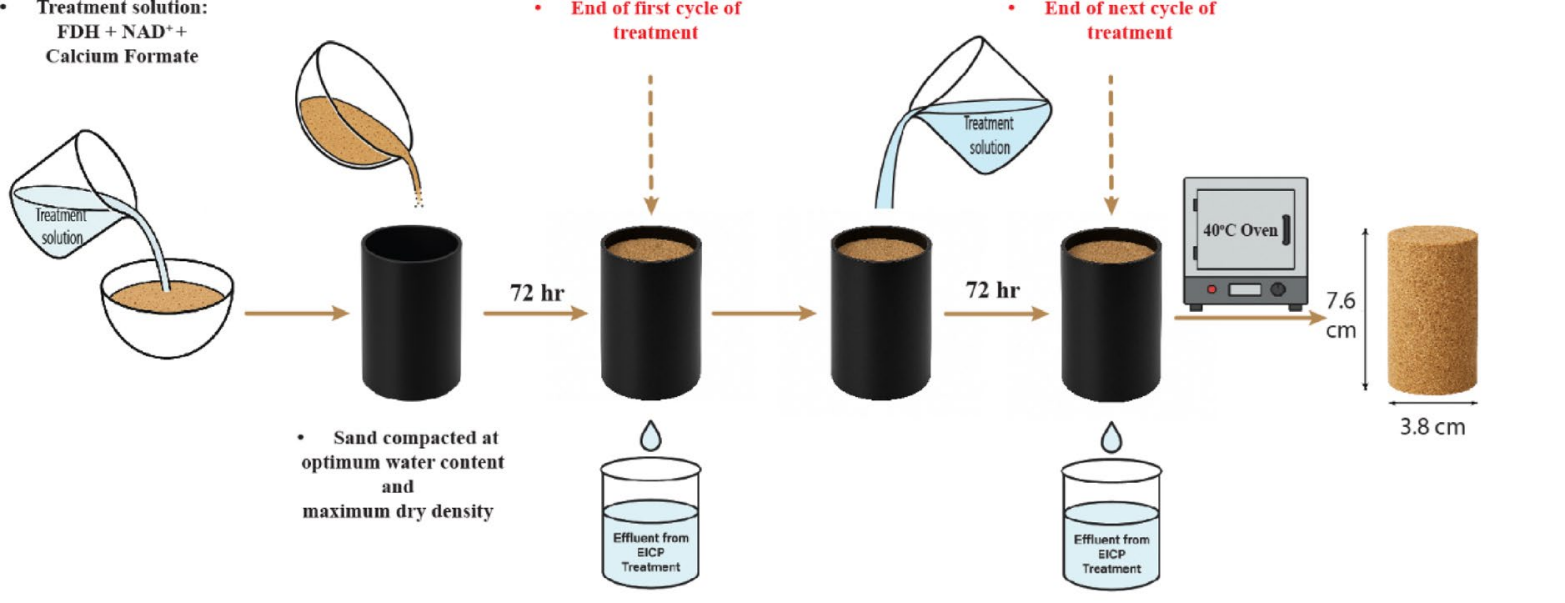
Sample preparation process.

In this study, two series of control tests were considered to validate the effectiveness of the non-ureolytic EICP method. These groups were untreated samples and samples treated with ureolytic EICP tests. The chemical and environmental conditions of the samples are indicated in Table 2. The preparation procedure and optimums for ureolytic EICP comply with previous studies to ease the comparison of results^46,48,71,74,75^. The sample preparation procedure is illustrated in Fig. 5.

**Table 2:** The treatment solution ingredients and the environmental conditions of the samples.

Non-ureolytic EICP samples							
Formate Dehydrogenase	NAD^+^	pH	Buffer (Potassium phosphate)	Calcium Formate	Curing time	Temperature	
75 mg/L (activity 1000 U/g)	290 g/L	7.6	100 mM	30 and50 g/L	72 h	25 °C	
Control 1: Ureolytic EICP samples							
Urease Enzyme	urea	pH	Buffer (Potassium phosphate)	Calcium chloride	Curing time	Temperature	References for compositions
3 g/L (activity 3500 U/g)	60 g/L	7.6	100 mM	95 g/L	72 h	25 °C	Almajed et al. 2018^71^
Control 2: Sand samples treated with control solutions (containing calcium source and lacking enzyme)							
Enzyme	NAD^+^	pH	Buffer (Potassium phosphate)	Calcium Formate	Curing time	Temperature	
Not used	290 g/L	7.6	100 mM	30 and50 g/L	72 h	25 °C	

### Unconfined compressive strength (UCS) tests

As mentioned in the previous section, UCS tests were conducted after the curing time. The UCS test is one of the most straightforward and standardized methods for measuring the mechanical strength of samples, indicating the efficiency of various soil improvement techniques. Furthermore, the test is widely used. Therefore, the results are comparable with those of previous studies. This test was conducted in dry and unconsolidated undrained (UU) conditions, following ASTM D2166, with a 1 mm/min strain rate. The test results were used to evaluate this method’s effectiveness in enhancing the treated soil’s strength and stability. The peak value of axial stress obtained from the UCS tests represented the mechanical strength (q*u*). The Secant Modulus (*Ef*) was determined by calculating the ratio of the peak strength (q*u*) to the corresponding strain at failure (*εf*). Additionally, E50, the secant stiffness modulus corresponding to 50% of the ultimate deviatoric stress, was also determined.

After completing the UCS test, the mass of calcium carbonate precipitated in each specimen was determined using a calcimeter test following each treatment cycle. In this study, a Bernard calcimeter was employed to measure the precipitation of CaCO_3_, utilizing a procedure similar to what was mentioned in the previous sections.

### Microfabric and physicochemical analysis

Microfabric analysis of soil samples treated with non-ureolytic EICP provides insights into the mechanisms contributing to the strength enhancement. The induced changes in soil porosity, as well as the particle-to-particle bonding, can be investigated through this analysis. Consequently, to scrutinize the microstructure of the samples, Scanning Electron Microscopy (SEM), X-ray Diffraction (XRD), and Fourier Transform Infrared Spectroscopy (FTIR) were employed. SEM was conducted using a TESCAN VEGA3 (TESCAN, Czech Republic). XRD and FTIR were performed on a D8 ADVANCE and a Tensor II (Bruker, Germany), respectively, at the central laboratory of Shiraz University.

## Results and discussion

### Optimization of environmental conditions and compositions for FDH

#### Optimization of environmental conditions

**Fig. 6:**
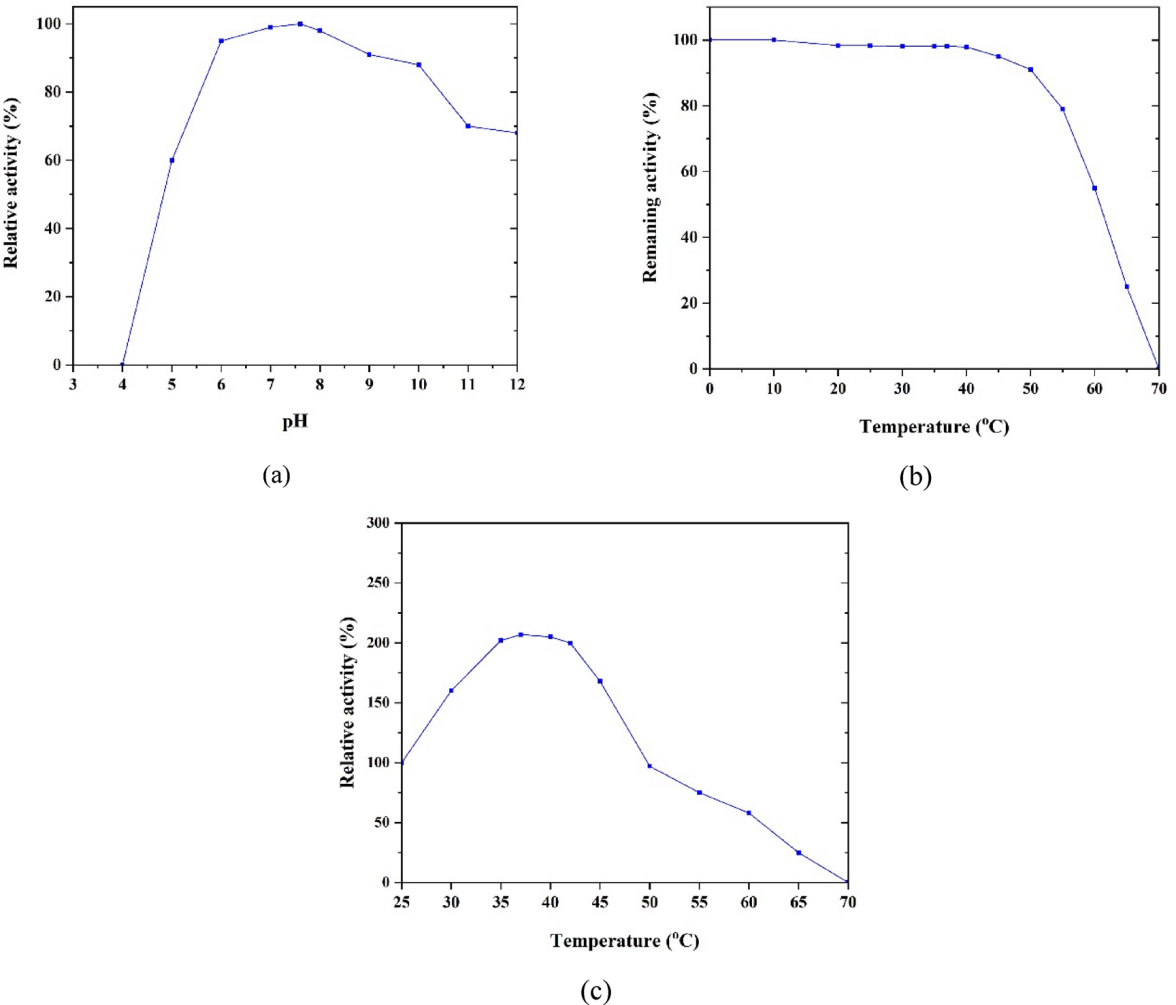
Effects of pH on the stability (**a**), remaining activity (stability) after 20 min at different temperatures (**b**), and relative activity at different temperatures (**c**).

Figure 6 illustrates the effect of pH and temperature on the stability and activity of FDH. The relative activity at different temperatures and pH values was determined by referencing measurements against optimal conditions under standard conditions. All the results were an average of three individual measurements. The enzyme activity is normalized to activity at 25 °C, and the data are shown against temperature and pH in Fig. 6 to determine the optimal conditions for FDH activity.

As depicted in Fig. 6, the maximum activity of FDH was observed at approximately 37 °C and a pH level of around 7.6. The enzyme is stable in a wide range of pH and temperature. The pH ranges where enzyme activity is highest correspond to the normal pH levels of soil and building materials, specifically 6 to 9. The stability between pH 10 and 12 is good but less efficient. The activity of the FDH enzyme remains at 91% after exposure to 50 °C. Exposure to temperatures above 50 °C decreases enzyme activity, yet it retains above 50% of its activity at 60 °C. However, at 70 °C, the enzyme exhibits no activity. Therefore, the activity and stability of this enzyme in harsh conditions are acceptable, making it suitable for use in a wide range of construction and building materials. Some FDHs (extracted from sources other than *Candida boidinii*, considered in this study) have been identified (e.g., *Ancylobacter aquaticus* FDH) that possess higher formate oxidation activity; nevertheless, they lack stability, making them unsuitable replacements for use in engineering applications^67,68^.

#### Optimization of required compositions

**Fig. 7:**
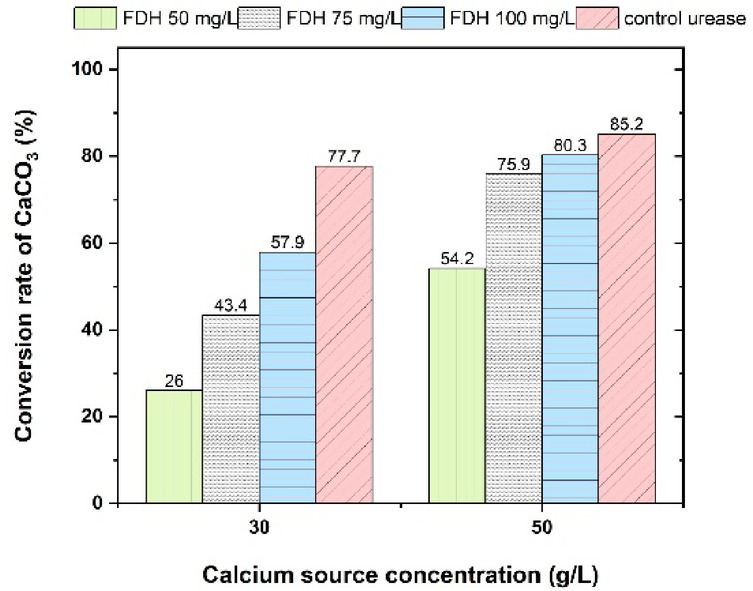
Conversion rate of CaCO_3_ in the tube test at different calcium sources and enzyme concentrations (for FDH-driven EICP, the calcium and carbon source is calcium formate, and for the control urease-based EICP, the calcium source is calcium chloride).

Figure 7 shows the CaCO_3_ conversion rate in the tube test. As illustrated in Fig. 7, increasing the calcium formate concentration enhances the amount of precipitated CaCO_3_. As illustrated in Fig. 7, after 72 h, at a concentration of 30 g/L of calcium formate, the difference in CaCO_3_ precipitation between 75 mg/L and 100 mg/L of the enzyme is significant. Increasing the calcium formate concentration to 50 g/L makes the difference between 75 and 100 mg/L less evident. Consequently, after 72 h, the most efficient combination of enzyme and calcium formate concentrations, balancing performance and cost, was found to be 50 g/L of calcium formate and 75 mg/L of the enzyme (FDH) (i.e., the lower enzyme concentration). Further increases in either component produced only marginal improvements in precipitation while significantly increasing the overall cost. Therefore, the treatment solution consisted of calcium formate at a concentration of 50 g/L, FDH enzyme at 75 mg/L, NAD⁺ at 290 g/L, and 100 mM potassium phosphate at a pH of 7.6 and 25 °C, which was selected for the UCS sample preparation. By comparing the results with the control urease-based EICP, non-ureolytic EICP (at the optimized reagent concentrations) can work approximately as efficiently as the control ureolytic EICP. Considering that this pathway does not produce any harmful byproducts, this method is deemed suitable for further investigation and potential application.

### Unconfined compressive strength test results

**Fig. 8:**
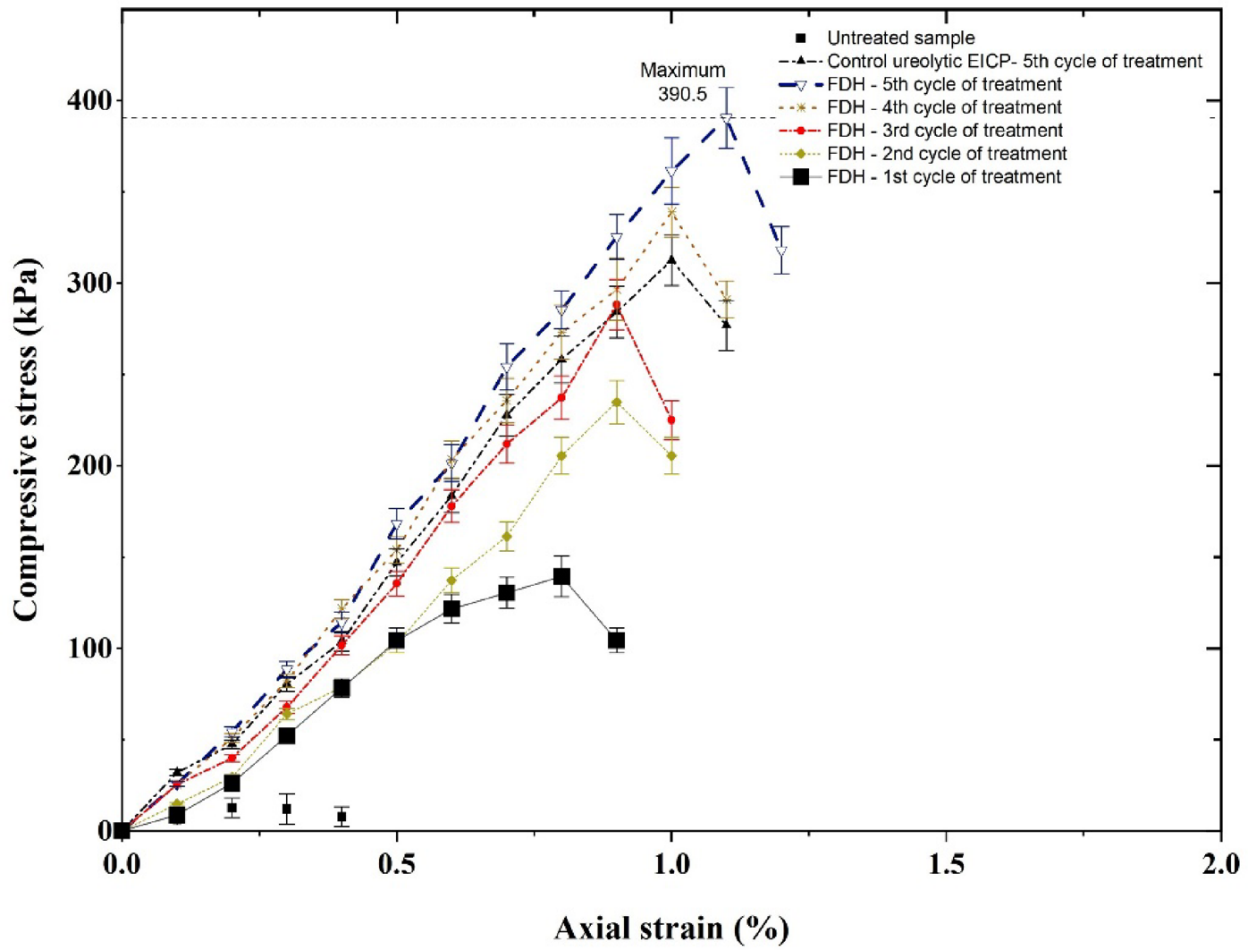
Stress-strain curves obtained from UCS tests on samples treated with non-ureolytic EICP.

Compressive stress versus axial strain results obtained for nonureolytic EICP (after 1 to 5 treatment cycles) are presented in Fig. 8. As shown in Fig. 8, increasing the treatment cycles increased the samples’ peak strength. This is attributed to the formation of calcium carbonate crystals that bond soil particles to each other and strengthen the soil matrix. The peak strength of the soil treated with 75 mg/L FDH and Calcium Formate 50 g/L after 5 treatment cycles reached a peak of 390.5 kPa. This demonstrates a remarkable 31-fold increase over the initial strength. Consequently, the peak strength in samples treated with enzymes was significantly higher than that of untreated samples. Although the control ureolytic EICP samples show a peak strength of 312 kPa, this is less than the strength gained by the sample treated with nonureolytic EICP at the same number of cycles.

The control samples did not show an increase in strength. This indicates that the strength improvements in the treatment samples were caused by calcium carbonate precipitation via the non-ureolytic EICP process. This precipitation raises bonding among soil particles and reduces porosity.

**Fig. 9:**
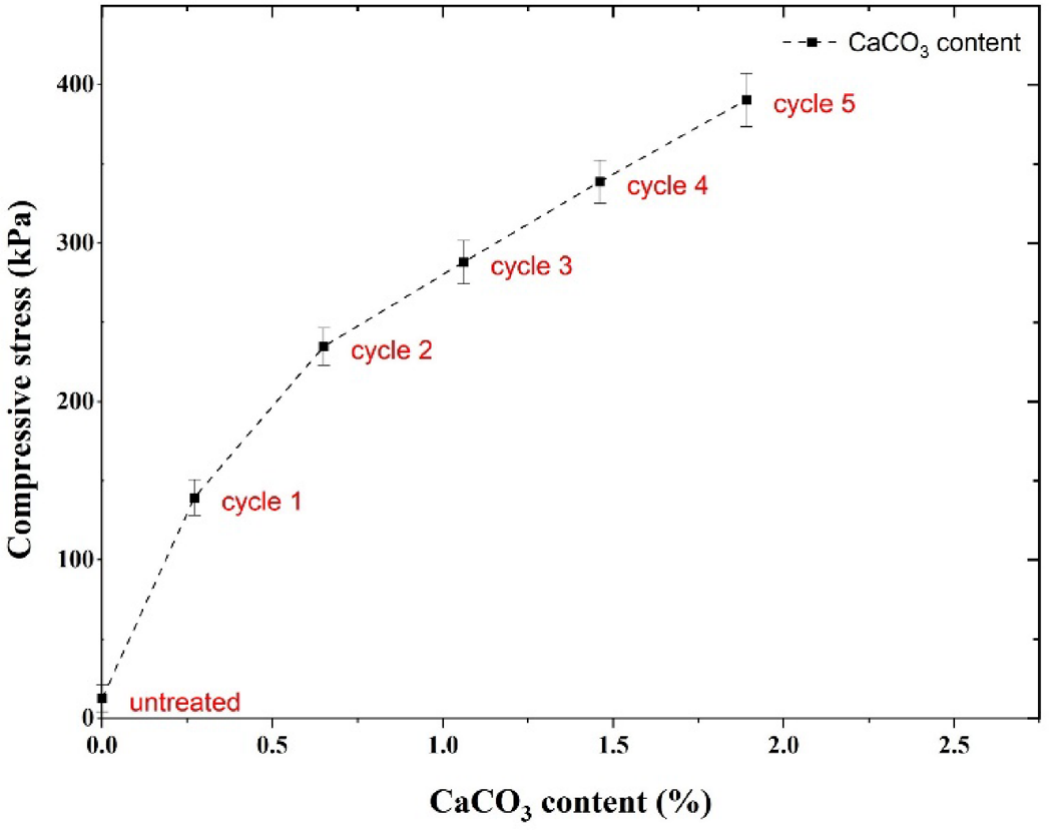
Maximum compressive stress versus CaCO_3_ content for samples treated with 1 to 5 cycles of non-ureolytic EICP.

Figure 9 illustrates the variation in compressive strength with increasing calcium content. As the number of treatment cycles increases, the calcium carbonate content increases; however, the increase in compressive strength is less pronounced at high treatment cycles (beyond the 3rd treatment cycle). It seems that preliminary precipitations form an effective calcite bridge between grains, and further precipitation may precipitate on already-formed crystals, forming peripheral precipitations, which are less effective in the mechanical bonding of the grains. The suggested mechanisms would, however, need evidence from sophisticated imaging studies such as 3D micro-computed tomography imaging in future studies.

The results of measuring the calcium carbonate content of the samples using a calcimeter are presented in Table 3. As can be seen, only 0.27% of calcium carbonate precipitated after one treatment cycle. This precipitation increased with the number of cycles. By the fifth cycle of treatment, it reaches 1.89%, which can significantly increase the strength of the samples.

**Table 3:** Results of the calcimeter test of non-ureolytic EICP treatment of soil samples used in this study.

Cycle number	CaCO_3_ (%)	CaCO_3_ (g)	q_u_ (kPa)
1	0.27	0.405	139.41
2	0.65	0.975	234.74
3	1.06	1.59	288.10
4	1.46	2.19	338.79
5	1.89	2.84	390.50

**Fig. 10:**
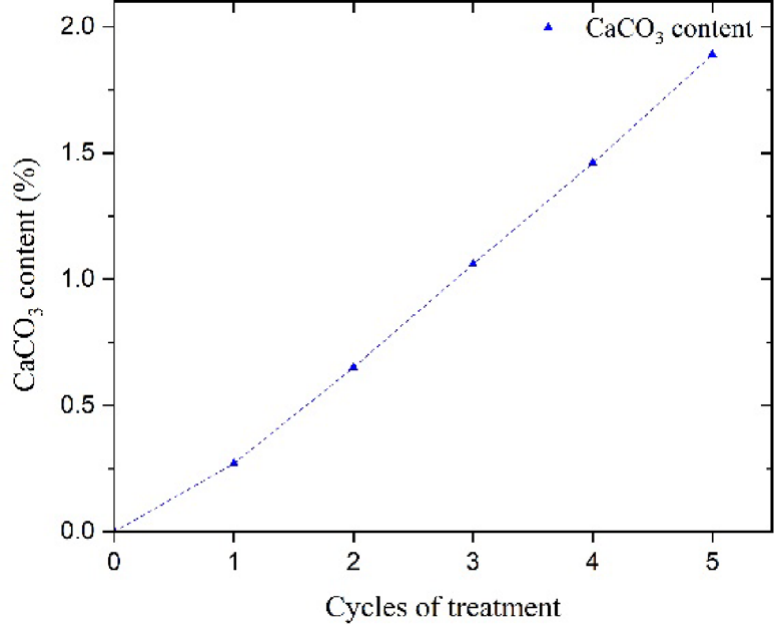
Effect of cycles of non-ureolytic EICP treatment on samples’ CaCO_3_ content.

**Fig. 11:**
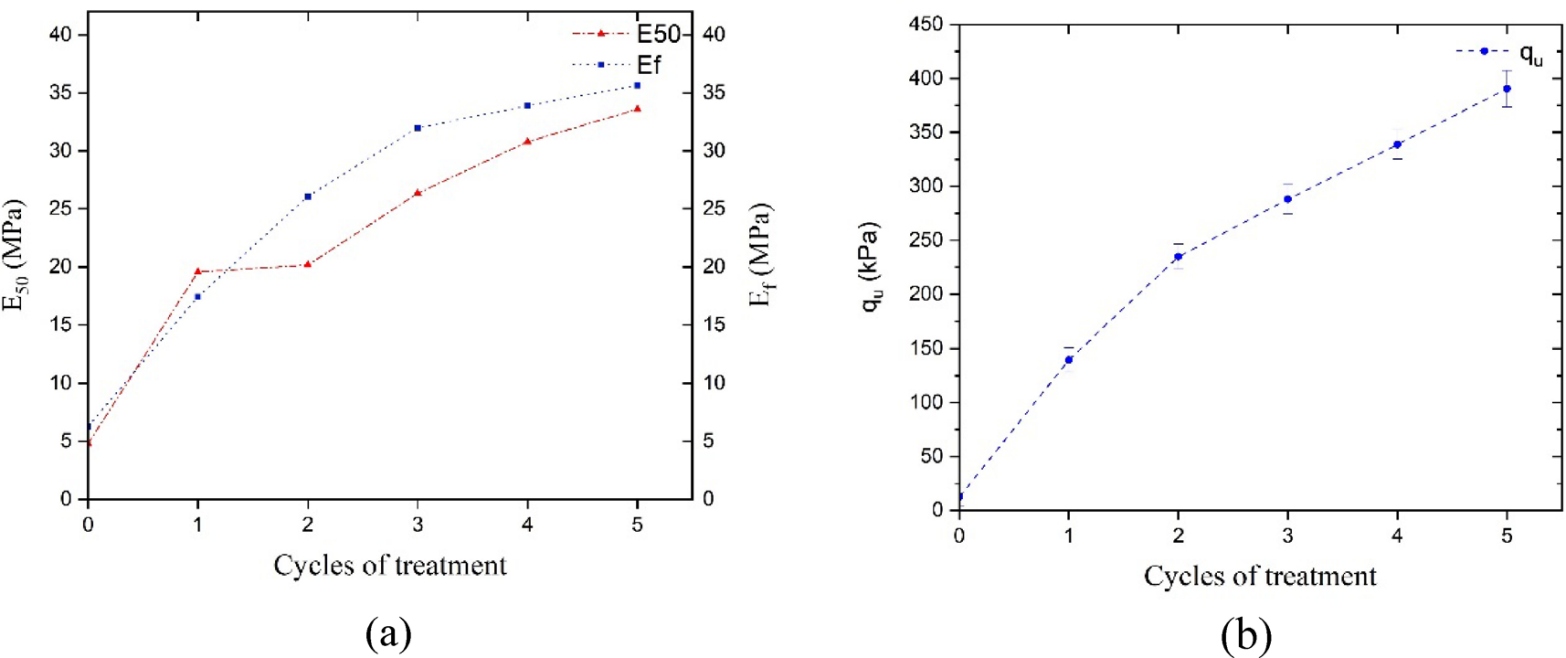
Effect of cycles of non-ureolytic EICP treatment on samples’ (**a**) E_50_ and E_f_ (**b**) q_u_.

Figures 10 and 11 illustrate the effect of treatment cycles on the modulus of elasticity and compressive strength. As can be seen, surpassing the 2nd cycle, the increase in modulus and strength is less steep and pronounced. Notably, this change in slope is more pronounced in E_f_ values compared to q_u_ results, indicating that the contribution of additional cycles to stiffness at failure (E_f_) is less noticeable at higher numbers of cycles. A treatment scenario, up to 3 cycles, at the considered experimental conditions, would be instrumental in achieving the E_50_ value of around 25 MPa and E_f_ of 32 MPa, an almost six-fold increase in stiffness.

### Micro-scale characteristics

**Fig. 12:**
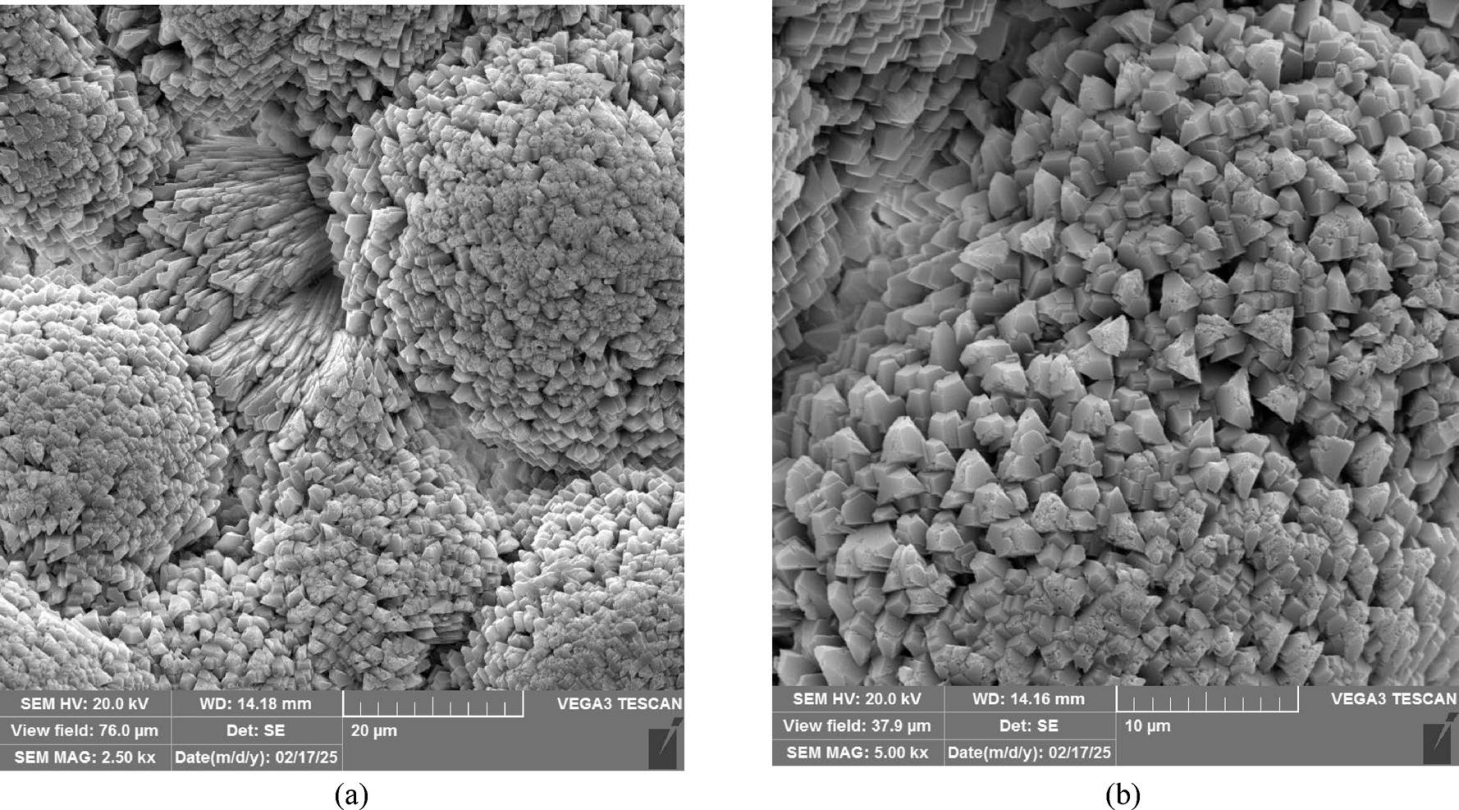
SEM images of calcium carbonate (CaCO₃) precipitated through non-ureolytic EICP using FDH. The crystals exhibit a rhombohedral morphology, characteristic of the calcite polymorph. (**a**) 2.5k× magnification, (**b**) 5k× magnification.

The effectiveness of EICP treatment depends on the precipitation of calcium carbonate within the soil pore space and particle agglomeration. This microfabric alteration improves the mechanical properties of the soil. Therefore, it is essential to understand the characteristics of the precipitates. These characteristics are crystal size, shape, and spatial distribution^76^. This section uses SEM, XRD, and FTIR results to discuss the factors influencing crystal characteristics, including pH and the concentrations of its constituents. Figure 12 shows the SEM image of the precipitated calcium carbonate by the FDH Enzyme.

**Fig. 13:**
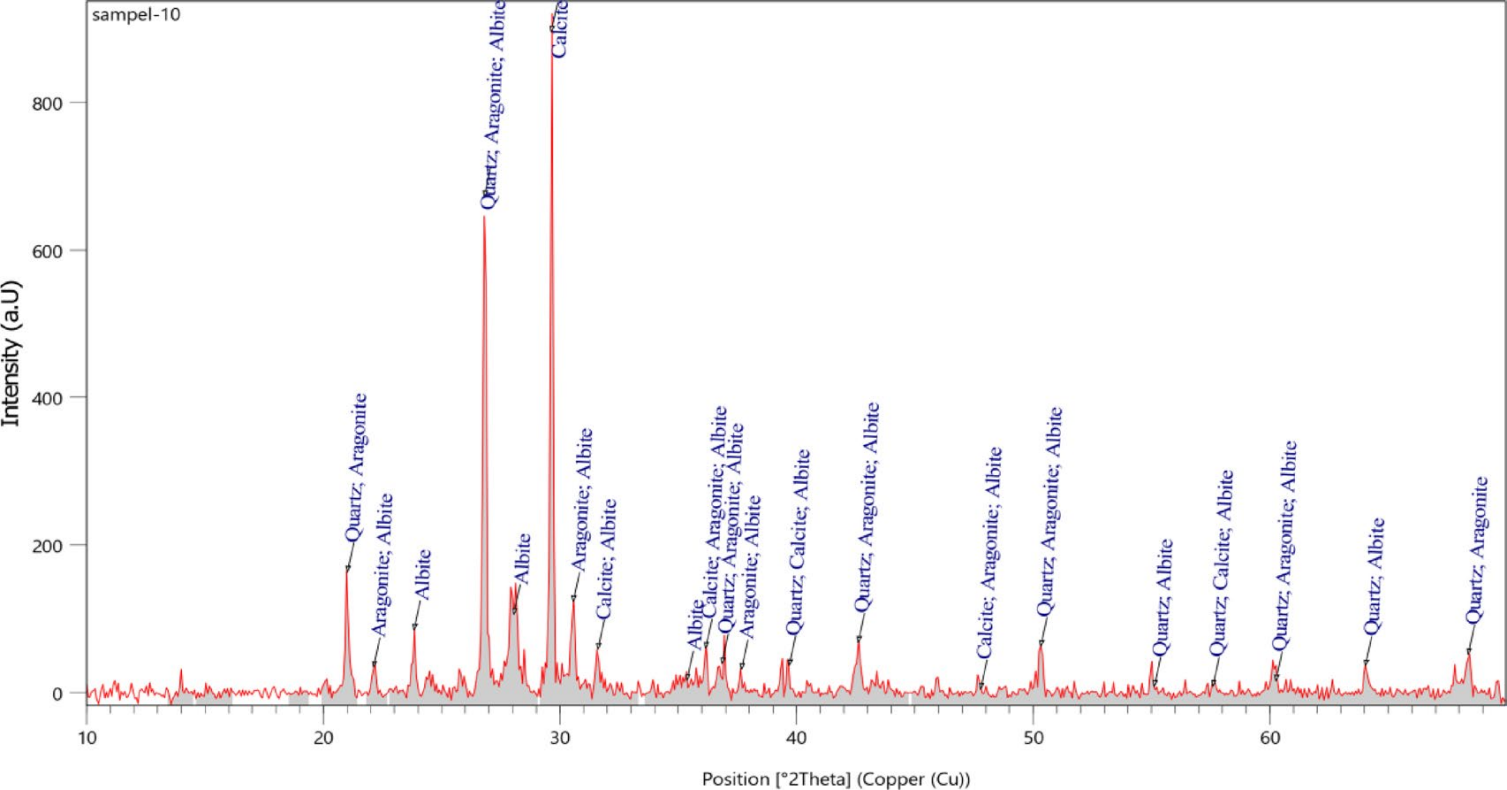
XRD spectra of the non-ureolytic EICP-treated Samples.

The crystals exhibit mostly rhombohedral morphology, indicative of the calcite polymorph. Although some crystals appear approximately cubic, this is consistent with the calcite crystal shape when viewed along specific crystallographic orientations. Rhombohedral calcite crystals with well-developed and distinct consolidation are the most suitable polymorph of CaCO_3_ for geotechnical applications^77^. The XRD spectra of samples treated with non-ureolytic EICP are shown in Fig. 13. The XRD patterns obtained in this study were compared with reference crystallographic data obtained from the Crystallography Open Database (COD). As indicated, calcite (COD database code: 9016706) is the predominant mineral. When narrowing the 2θ range to around 29–30°, the predominant 104 calcite peak, which corresponds to the (1 0 4) crystal plane, becomes evident. Further focusing on the 43–49° range reveals the expected 202, 204, and 108 calcite peaks, which correspond to the (2 0 2), (2 0 4), and (1 0 8) crystal planes, respectively. Narrowing to the 39–40° range highlights the 113-calcite peak, which relates to the (1 1 3) crystal plane^78,79^. The peaks relating to aragonite (COD database code: 9014833) appear around 24° and 27°, indicating that aragonite is a significant secondary phase. Vaterite (COD database code: 9013565) is also detected around 32.8° but with a lower intensity than calcite and aragonite.

**Fig. 14:**
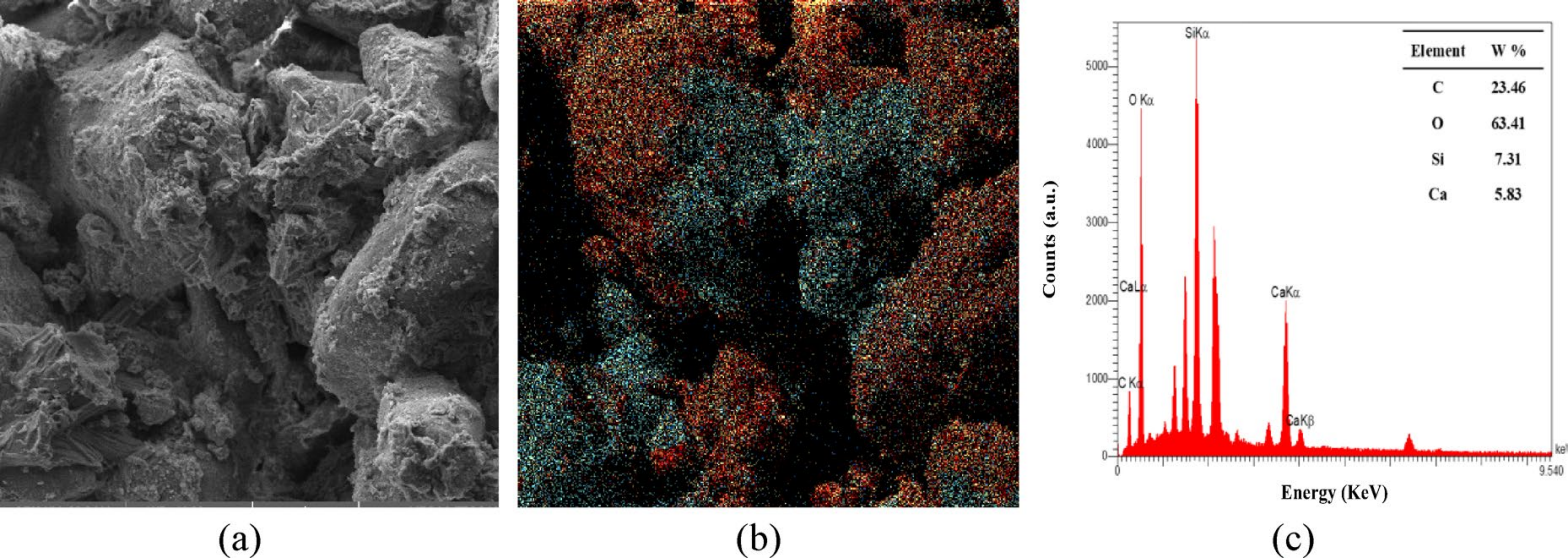
(**a**) EDX analysis of the samples treated with non-ureolytic EICP, (**a**) the SEM image, (**b**) the SEM image with combined spectra, the cyan colored region indicating the precipitates, (**c**) the EDX analysis result.

Figure 14 shows the SEM and EDX spectra of the non-ureolytic EICP-treated sample. As the figure indicates, CaCO_3_ precipitation covers the grains’ surfaces and occupies the voids between grains. The precipitates exhibit a compact and organized structure, indicating that calcium carbonate has been successfully deposited throughout the pore spaces and along grain boundaries. These combined effects can bind soil particles, fill pore space, and accordingly reduce porosity and increase soil agglomeration. As a result, an increase in soil strength is achieved, as observed earlier.

**Fig. 15:**
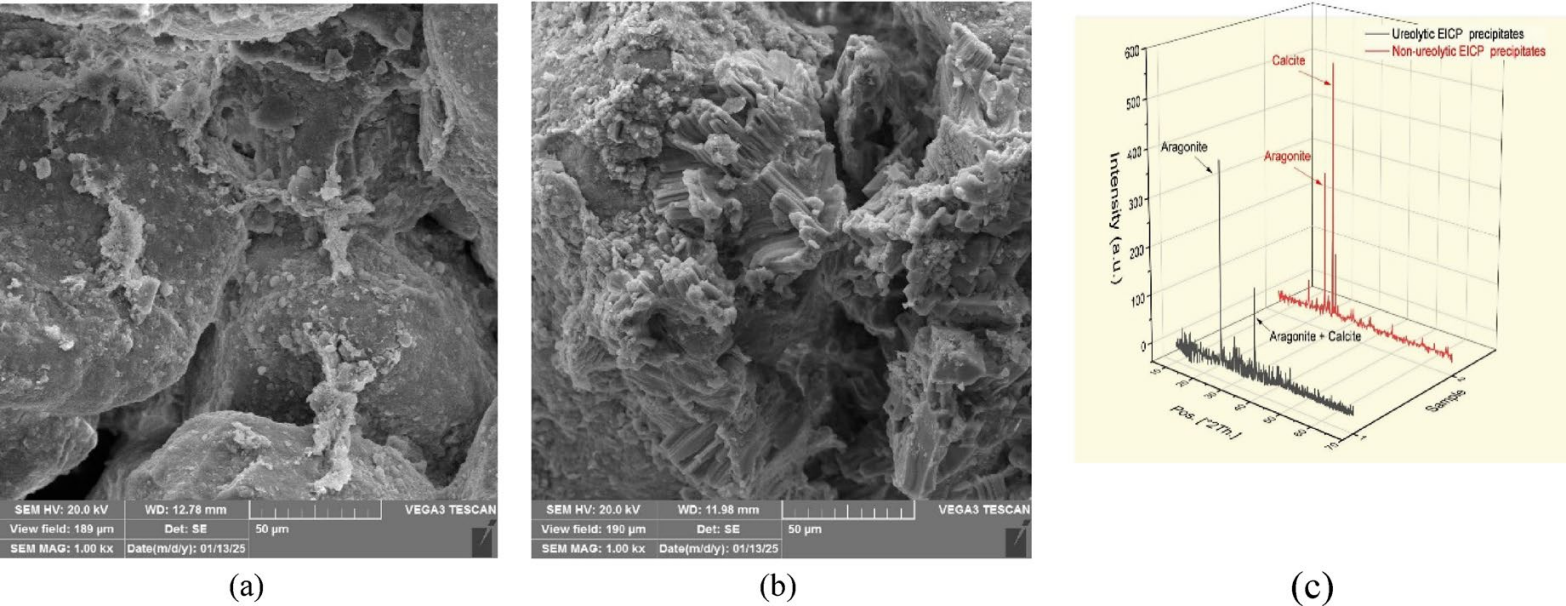
(**a**) SEM of the samples treated with ureolytic EICP and (**b**) non-ureolytic EICP; (**c**) XRD of the precipitates.

The SEM of ureolytic and non-ureolytic EICP-treated samples and XRD spectra of precipitates are shown in Fig. 15. Figure 15a presents the SEM micrograph of the control sample treated with ureolytic EICP, while Fig. 15b displays the non-ureolytic EICP-treated sample. As observed, the ureolytic EICP sample contains less compacted and loosely arranged CaCO_3_ precipitates. Conversely, the non-ureolytic EICP sample has dense and well-structured crystals that effectively cover the pore spaces and grain surface.

The XRD analysis reveals that the CaCO_3_ crystals precipitated in non-ureolytic EICP were mainly calcite and aragonite. Vaterite was also observed in smaller quantities, which affects soil porosity. However, the conventional EICP treatment solution produced significantly more vaterite crystals^5^. The rapid rate of calcium carbonate precipitation in traditional EICP contributes to forming more vaterite crystals^5,77^. Aragonite and vaterite polymorphs were less favored in the EICP treatment than calcite. Aragonite and vaterite exhibit irregular or spherical shapes. Their shape limits their capability for nucleation and structural reinforcement^70^. A governing factor in the achieved mechanical strength would be the density and integrity of precipitates. The denser the precipitates form, the higher the achieved strength will be.

Therefore, the less compacted precipitates in ureolytic EICP impact the soil’s mechanical properties and can explain the weaker mechanical properties. Overall, the SEM images confirm that crystal morphology and packing density play a key role in the efficiency of EICP treatments.

**Fig. 16:**
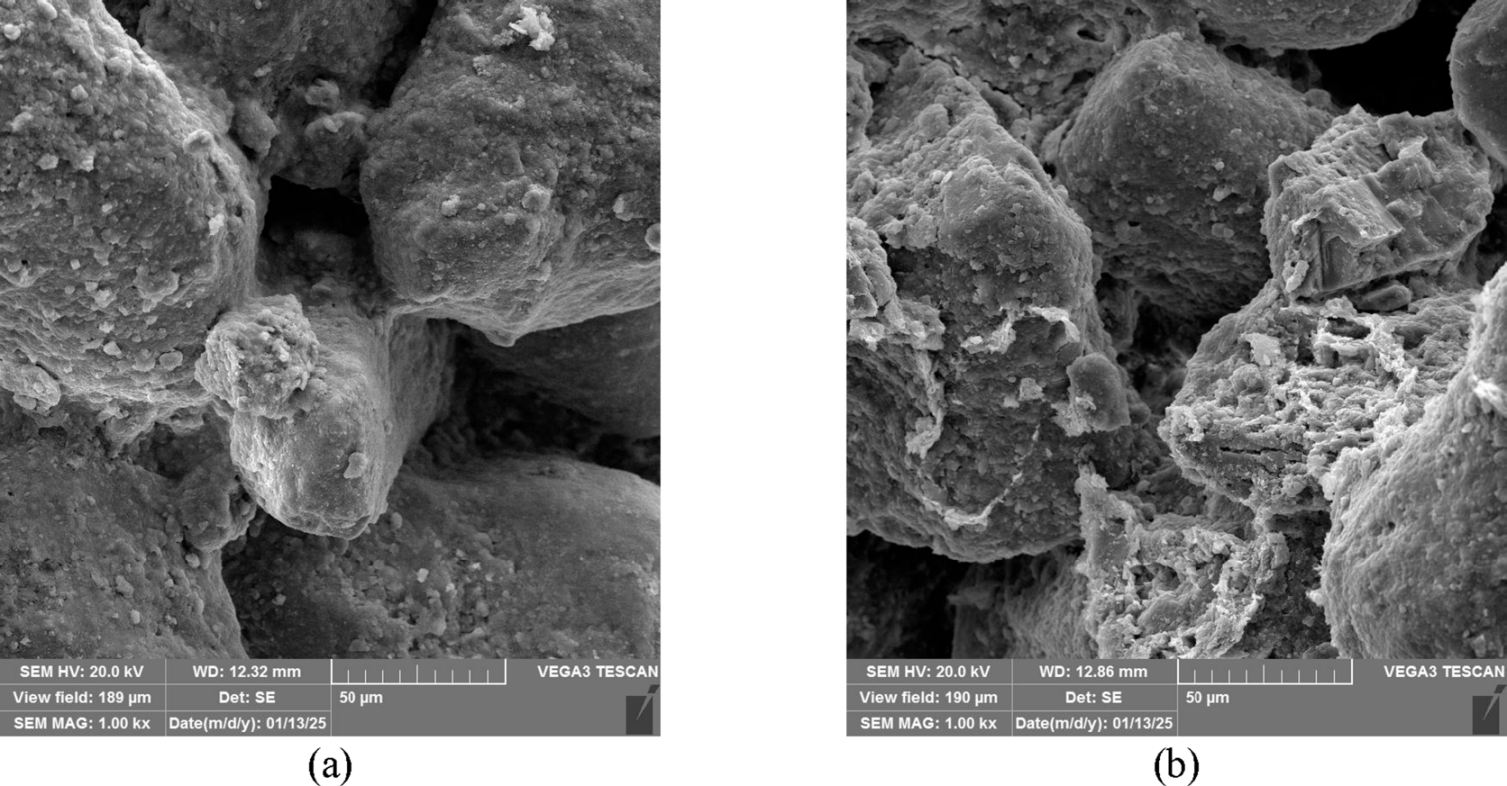
SEM of the samples treated with (**a**) EICP with FDH after one cycle of treatment (**b**) EICP with FDH after 5 cycles of treatment.

The SEM images of the samples treated with FDH Enzyme after 1 and 5 cycles of treatment are shown in Fig. 16. As can be seen, the number and thickness of soil bonds increased with the number of treatment cycles. These precipitates bind soil particles together and can potentially decrease the soil porosity and permeability, as reported in the previous studies^80^. The reduction in effluent flow rate can indicate a decrease in permeability. Figures A.1 to A.4 in Appendix A (Supplementary Material) present analyses conducted on effluent. The change in its flow rate is discussed in Figure A.4, clearly signifying this reduction. The strength increase was also observed in the UCS test results reported earlier.

**Fig. 17:**
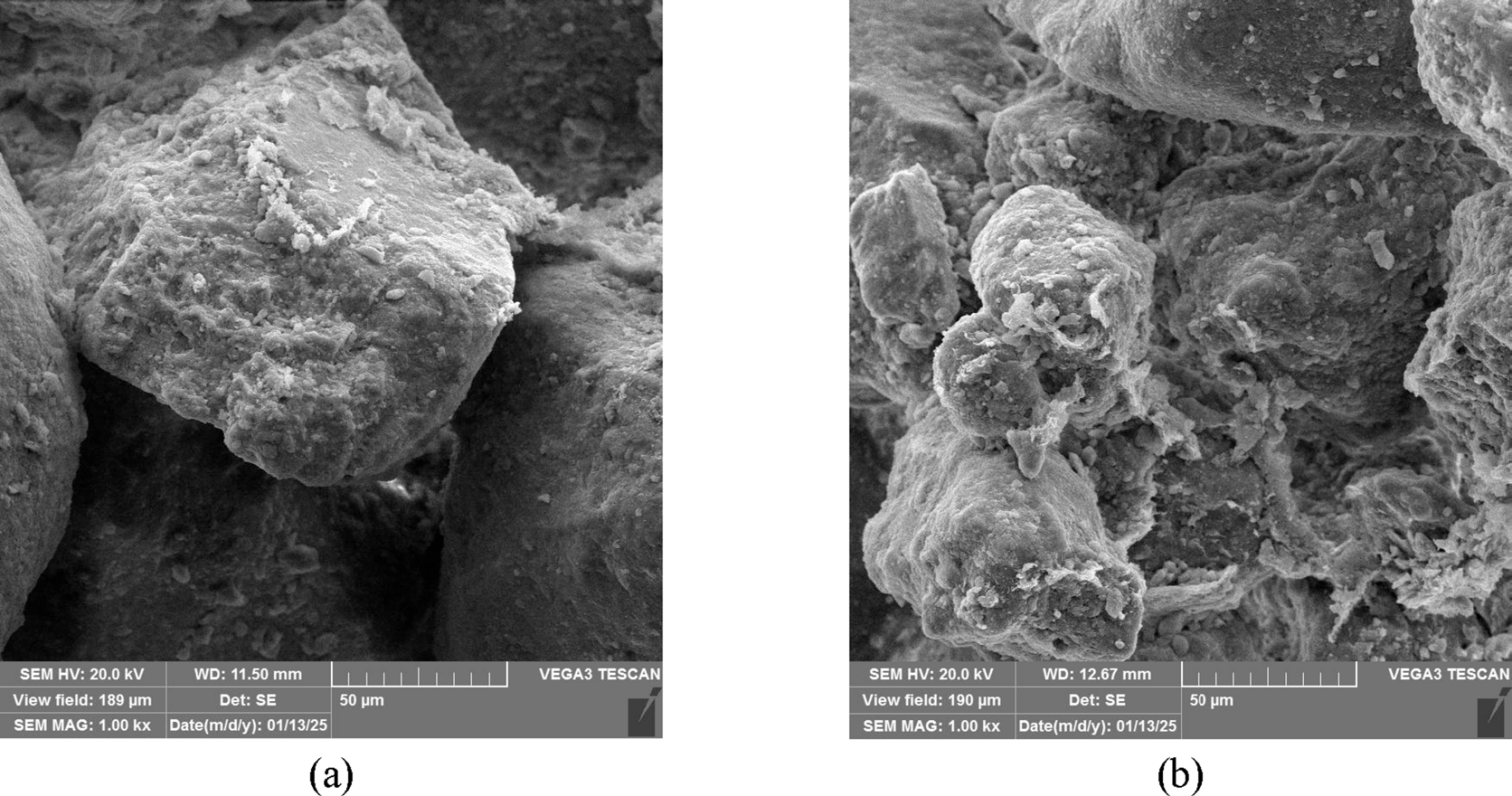
SEM of the samples treated after 1 cycle of treatment with non-ureolytic EICP with (a) calcium formate at 30 g/L (b) calcium formate at 50 g/L

The SEM images of the samples treated with the FDH enzyme at different calcium formate concentrations are shown in Fig. 17. Figure 17a exhibits limited calcium carbonate crystals on the sand particle surface and at the interparticle contacts of sand grains. As revealed by Fig. 17b, in samples treated with calcium formate concentrated at 50 g/L, a higher calcium content in the soil leads to more aggregation, facilitating stabilization. Hence, increasing the amount of calcium formate to 50 g/L enhances soil aggregation, particle-to-particle bonding, and coating the particle surface. Consequently, the soil’s strength increases.

**Fig. 18:**
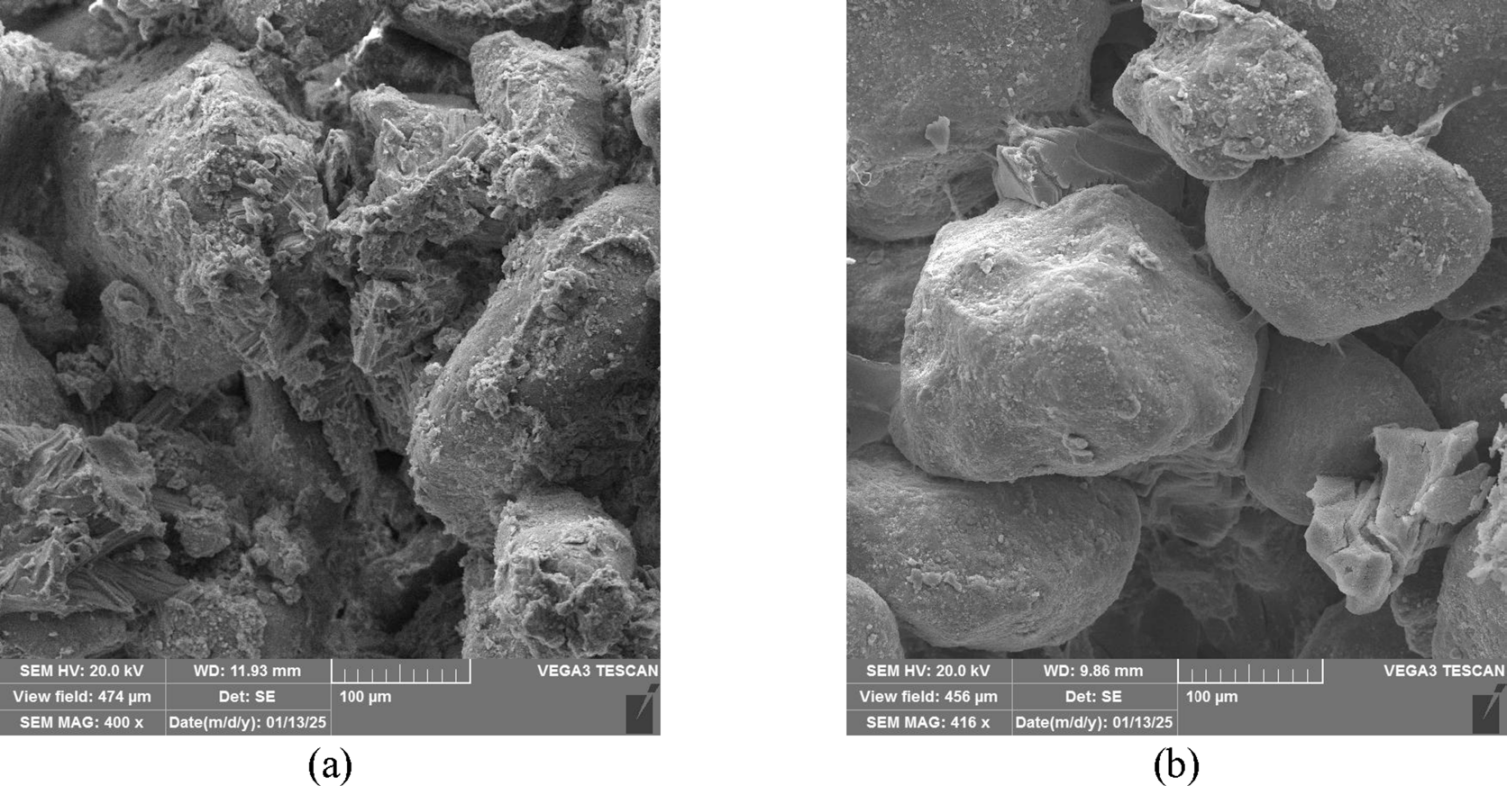
SEM of the samples treated at (**a**) pH 7.6 (**b**) pH 11.

Figure 18 presents the SEM images of the samples treated with non-ureolytic EICP at pH of 7.6 and 11. The image indicates that the crystal shape changes by varying the pH level. The pH change alters the morphology and size of the crystals^81^. At 7.6 pH, the speed of the crystal precipitation is enough for the compacted and well-structured calcite to precipitate. The change in precipitation ratio over time at pH levels of 7.6 and 11 is presented and analyzed in Appendix B (Figure B, Supplementary Material), indicating a rapid initial reaction rate at high pH levels. At excessively high pH levels, the rapid reaction leads to immediate supersaturation and the rapid precipitation of CaCO₃. This results in numerous nucleation sites and hinders the growth of well-ordered, compact crystals. Consequently, the precipitates exhibit a loosely packed, highly porous structure. This limits the effectiveness of soil grains’ binding and strength improvement. In addition, in high pH conditions, the stability of enzymes is lower; thus, with every cycle, the enzyme is denatured slightly, and the efficiency decreases^81^.

**Fig. 19:**
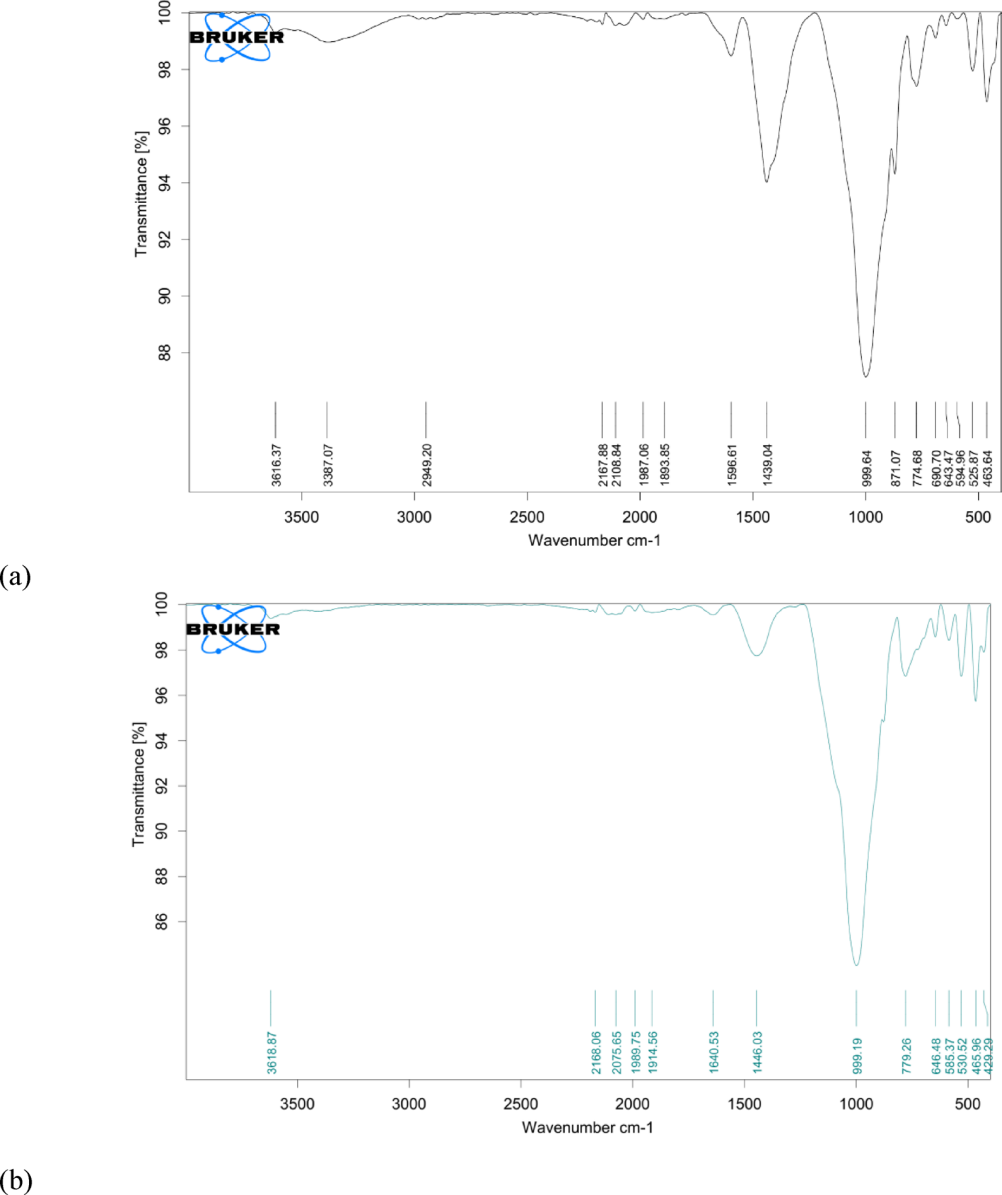
FTIR spectra of the samples treated with (**a**) non-ureolytic EICP and (**b**) ureolytic EICP.

Figure 19 illustrates the FTIR spectra of the samples treated with non-ureolytic and ureolytic EICP. The non-ureolytic spectra display a relatively sharp peak at approximately 1439 cm⁻¹; its sharpness is characteristic of calcite and aragonite. Furthermore, a relatively sharp peak at around 871 cm^- 1^ and a sharp band at 774 cm^- 1^ indicate some calcite formation^79^. The peaks around 994 cm^- 1^ belong to the quartz and soil properties, and a light peak at 3387 cm^- 1^ belongs to the hydroxyl bond of water within the soil^71,82^. These results indicate the composition of the interparticle bonds formed by the precipitation of non-ureolytic EICP, affecting the treated samples’ mechanical properties. The FTIR spectra of ureolytic EICP display a strong absorption peak around 999 cm⁻¹, along with moderate peaks in the 1400–1470 cm⁻¹ range associated with carbonate stretching. This pattern is characteristic of the vaterite polymorph of calcium carbonate, in addition to quartz and soil properties. The absence of the distinct 713 cm⁻¹ peak suggests that calcite is not the dominant phase. These results indicate that vaterite was the primary form of CaCO₃ precipitated during the EICP process.

Overall, the SEM images, XRD, and FTIR spectra indicate CaCO_3_ precipitation as calcite and aragonite polymorphs. The precipitated CaCO_3_ has bonded sand grains, and these bonds can provide reasonable mechanical strength. The higher strength of the non-ureolytic EICP process is caused by CaCO_3_ crystals that were closely packed, attached to the sand particles, and well distributed. In contrast, the CaCO_3_ crystals from the control ureolytic EICP process were formed by precipitates that were much more scattered and not well attached to the sand particles.

### Discussion of non-ureolytic EICP applications and challenges

The EICP method has emerged as a sustainable and innovative method for soil stabilization. Cement production alone is responsible for 5–8% of global CO₂ emissions7, whereas EICP relies on bio-based materials and enzymes, significantly reducing its environmental impact. The EICP precipitation can bind soil particles, enhance strength and stiffness, and decrease permeability while maintaining a lower carbon footprint than conventional methods^76,83–85^. The conventional ureolytic EICP pathway faces challenges in implementation, including managing ammonium byproducts and maintaining urease enzyme stability under field conditions. Addressing these challenges will be crucial for the practical applications of this method^84,85^.

This study offers a sustainable and environmentally friendly alternative to traditional ureolytic EICP. This method addresses challenges faced by the current EICP variant. The FDH enzyme does not produce ammonium byproducts because the biochemical pathway differs from urea hydrolysis. This method does not require the use of urea in the process, thereby circumventing the associated environmental problems. Additionally, the FDH remains stable under various environmental conditions. Hence, the stability and workability under field conditions can also be fully addressed.

The cost comparison of EICP treatment methods, considering enzyme type and pathway, is shown in Table 4. As can be seen, the non-ureolytic method results in an approximately similar price to urease-based or traditional EICP enzymes. Most notably, the absence of ammonium byproducts is a great advantage of the proposed nonureolytic-EICP. Furthermore, as a considerable portion of the cost in EICP techniques is related to the enzyme price, substituting the pure enzyme with a crude enzyme would make this method more efficient and cost-effective.

**Table 4:** Cost details of the enzyme solution for EICP treatments.

Stabilization method	Typical Dose per L solution^70^	Unit price^*^ (USD/g)	Total price (USD/L)
Non-ureolytic EICP (lab-grade)	75 mg/L	500–1000	37.5–55
Non-ureolytic EICP (crude-grade)	180 g/L	0.1	18
Urease-based EICP (lab-grade)^70^	300 mg/L	125.7–200	38.25-60
Urease-based EICP (crude-grade)^70^	180 g/L	0.08	14.4

* Prices gathered from the enzyme supplier Sigma Aldrich (https://www.sigmaaldrich.com)

To further reduce costs in non-ureolytic EICP, the effluent obtained after treating samples can be reused. As discussed in Appendix A (Supplementary Material), the effluent contains unreacted formate, NADH (produced via FDH activity), potentially residual FDH enzyme, and dissolved impurities. Standard microfiltration (0.45 μm) can remove suspended fines and soil particles while retaining soluble components that drain from the soil matrix, such as NADH and the residual FDH enzyme. This approach provides a sustainable and cost-effective method for reusing cofactors across multiple EICP cycles, thereby enhancing the feasibility of large-scale soil stabilization or biocementation processes. This procedure can be examined in future studies.

Despite its advantages, the proposed non-ureolytic EICP has specific limitations, including controlling supersaturation and ensuring uniform distribution in heterogeneous and fine-grained soils. Besides, researching potential sources of non-ureolytic crude enzymes and their applications, reducing cost for industrial-scale applications is highly recommended. Another future research topic could be investigating a mixing-based treatment method combined with higher concentrations of treatment solution ingredients for fine-grained soils. Additionally, the effect of this method on hydraulic soil properties and characteristics needs to be investigated through a comprehensive and detailed permeability and sedimentation test, which is crucial for future investigations. While recycling effluent is beneficial for cost reduction, this recycling may be challenging in field applications. In this study, the effluent was collected for further analysis but not reused. As indicated earlier, a set of chemical and physical characterization experiments were conducted on effluent, and the results are presented in Appendix A (Supplementary Material). The FTIR results of the effluent after the first and fifth cycles of treatment are shown in Figure A.1. The SEM and EDX results of the particles carried by effluent after the first and fifth cycles of treatment are presented in Figures A.2 and A.3. Figure A.4 indicates the change in effluent flow rate with the addition of treatment cycles. However, a more complete chemical analysis of the effluent in future studies is necessary to investigate its potential for reuse. The feasibility of field recycling of the effluent needs further studies. Furthermore, the long-term durability of treated soils under environmental stresses requires further investigation. Addressing these challenges through research and field trials will be necessary to advance this method for large-scale applications.

**Fig. 20:**
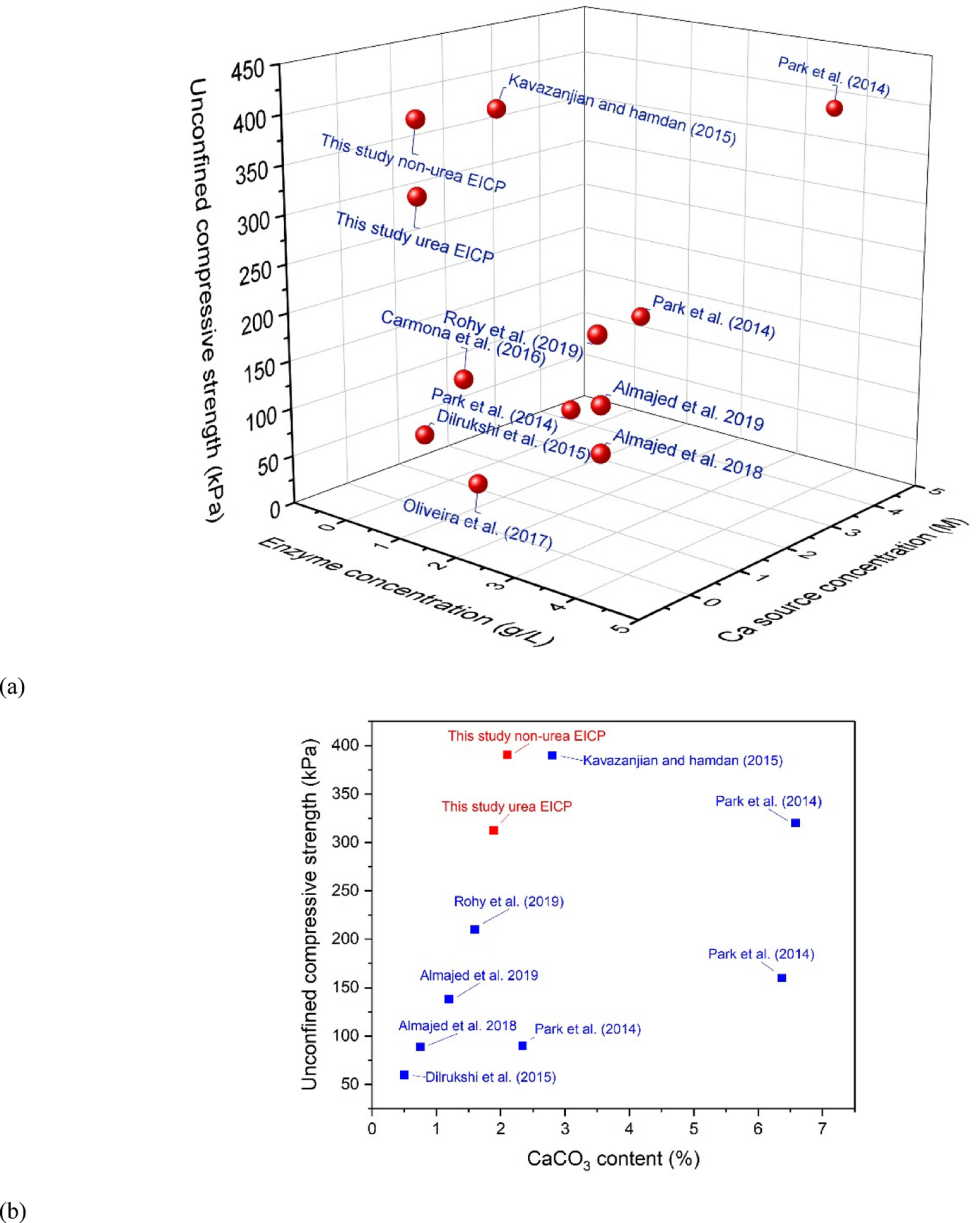
Comparison of UCS results of non-ureolytic EICP versus other conventional (ureolytic) EICP studies (**a**) By enzyme concentration and calcium source concentration (**b**) by CaCO_3_ content^31,48,71,75,86–91^.

In Figure 20, the UCS results of non-ureolytic EICP are compared with those from other EICP studies conducted under the same conditions, specifically examining the effect of calcium (Ca) source concentration, enzyme concentration, and CaCO_3_ content. As can be seen, at almost the same amount of precipitated calcium carbonate, the non-ureolytic EICP gives good strength results while posing fewer environmental concerns. This method yields sufficiently good soil improvement results, potentially a suitable alternative to the conventional EICP method, which produces an ammonium byproduct. Consequently, the non-ureolytic EICP represents a potentially transformative technology for geotechnical engineering and environmental preservation.

## Conclusions

Non-ureolytic enzyme-induced calcium carbonate precipitation (EICP) was investigated as an environmentally friendly method that does not produce harmful ammonium byproducts. This method utilizes the formate dehydrogenase (FDH) enzyme from Candida boidinii, calcium formate as a calcium source, potassium phosphate as a buffer, and NAD⁺ as a cofactor. The enzyme’s resistance to variations in pH and temperature was investigated. Cylindrical soil samples were treated under various conditions. Unconfined compressive strength (UCS) tests, calcium carbonate content measurements, and microstructural analyses (SEM, XRD, FTIR) were conducted. The major findings are as follows:

- The FDH enzyme maintained at least 90% activity within the pH range of 6–9 and at temperatures up to 50 °C. The maximum activity of FDH was noted at approximately 37 °C and a pH level of around 7.6.
- Soil strength improved significantly after treatment. UCS values increased 10-fold (139.41 kPa) after one treatment cycle and 31-fold (390.5 kPa) after five cycles. It achieved an E_50_ value of approximately 25 MPa and an E_f_ of 32 MPa, representing an almost six-fold increase in stiffness.
- After one treatment cycle, 0.27% of calcium carbonate precipitated. This precipitation increased with the number of cycles. By the fifth treatment cycle, it reached 1.89%, which can enhance the strength of the samples by about 31-fold.
- SEM, XRD, and FTIR analyses confirmed that calcite is the dominant crystal form, with some aragonite also observed. Both polymorphs of precipitated carbonate contribute to strength, though calcite has a more significant influence due to its (higher environmental stability) crystal shape.
- Very high pH levels (e.g., 11) can shift the precipitation crystal shape and concentration. At optimum pH, the precipitation fabric is compacted and well-structured. However, at high pH, the crystals no longer make a compact precipitation pattern and exhibit high porosity, low aggregation, and binding ability.

In conclusion, the proposed non-ureolytic EICP method demonstrates broad operational stability, enhances soil strength, and eliminates harmful byproducts. Its environmental compatibility, mechanical benefits, and potential for sustainable implementation make it suitable for future sustainable soil stabilization applications.

## Supplementary Information

Below is the link to the electronic supplementary material.

Supplementary Material 1

## Data Availability

The data that support the findings of this study are available from the corresponding author upon reasonable request.

## References

[1] Ellis, L. D., Badel, A. F., Chiang, M. L., Park, R. J. Y. & Chiang, Y. M. Toward electrochemical synthesis of cement—An electrolyzer-based process for decarbonating CaCO3 while producing useful gas streams. *Proc. Natl. Acad. Sci.* **117**, 12584–12591 (2020). 31527245 10.1073/pnas.1821673116 PMC7293631

[2] York, I. & Europe, I. Concrete needs to lose its colossal carbon footprint. *Nature* **597**, 593–594 (2021). 34584258 10.1038/d41586-021-02612-5

[3] Whiffin, V. S. *Microbial CaCO3 Precipitation for the Production of Biocement* (PhD diss, 2004).

[4] Dejong, J. T. et al. Ice Publishing,. Biogeochemical processes and geotechnical applications: progress, opportunities and challenges. In: *Bio-and chemo-mechanical processes in geotechnical engineering: géotechnique symposium in print 2013* 143–157 (2014).

[5] Ahenkorah, I., Rahman, M. M., Karim, M. R. & Beecham, S. Unconfined compressive strength of MICP and EICP treated sands subjected to cycles of wetting-drying, freezing-thawing and elevated temperature: experimental and EPR modelling. *J. Rock Mech. Geotech. Eng.* **15**, 1226–1247 (2023).

[6] Moravej, S., Habibagahi, G., Nikooee, E. & Niazi, A. Stabilization of dispersive soils by means of biological calcite precipitation. *Geoderma* **315**, 130–137 (2018).

[7] Deylaghian, S., Nikooee, E., Habibagahi, G. & Nagel, T. Inulin biopolymer as a novel material for sustainable soil stabilization. *Sci. Rep.* **14**, 31078 (2024). 39730738 10.1038/s41598-024-82289-8 PMC11680679

[8] Cappai, M. et al. Thermal properties of eco-friendly earthen materials stabilized with bio-based polymers: experimental data and modeling procedure for improving mix-design. *Materials* **17**, 1035 (2024). 38473506 10.3390/ma17051035 PMC10934699

[9] Niknam Safari Kouchi, E., Nikooee, E., Habibagahi, G., Niazi, A. & Nagel, T. The swelling characteristics of an unsaturated bio-cemented sand-bentonite mixture: analyzing the effect of bacterial concentration and suction. *Iranian J. Sci. Technol. Trans. Civil Engineering*, 1–21 (2025).

[10] Al Qabany, A., Soga, K. & Santamarina, C. Factors affecting efficiency of microbially induced calcite precipitation. *J. Geotech. GeoEnviron. Eng.* **138**, 992–1001 (2012).

[11] van Paassen, L. A., Ghose, R., van der Linden, T. J., van der Star, W. R. & van Loosdrecht, M. C. Quantifying biomediated ground improvement by ureolysis: large-scale biogrout experiment. *J. Geotech. GeoEnviron. Eng.* **136**, 1721–1728 (2010).

[12] Saffari, R., Nikooee, E., Habibagahi, G. & Van Genuchten, M. T. Effects of biological stabilization on the water retention properties of unsaturated soils. *J. Geotech. GeoEnviron. Eng.* **145**, 04019028 (2019).

[13] Sadjadi, M., Nikooee, E. & Habibagahi, G. Biological treatment of swelling soils using microbial calcite precipitation. *Unsaturated Soils: Res. Applications*, 917–922 (2014).

[14] DeJong, J. T., Fritzges, M. B. & Nüsslein, K. Microbially induced cementation to control sand response to undrained shear. *J. Geotech. GeoEnviron. Eng.* **132**, 1381–1392 (2006).

[15] Saffari, R., Habibagahi, G., Nikooee, E. & Niazi, A. Biological stabilization of a swelling fine-grained soil: the role of microstructural changes in the shear behavior. *Iran. J. Sci. Technol. Trans. Civil Eng.* **41**, 405–414 (2017).

[16] Sisakht, B., Nikooee, E., Habibagahi, G. & Niazi, A. Stabilisation of collapsible soils: a biological technique. (2015).

[17] Cardoso, R., Borges, I., Vieira, J., Duarte, S. O. & Monteiro, G. A. Interactions between clay minerals, bacteria growth and urease activity on biocementation of soils. *Appl. Clay Sci.* **240**, 106972 (2023).

[18] Afzali, S. F., Hemayati, M., Niazi, A. & Nikooee, E. Toward industrialization of microbially induced carbonate precipitation for wind erosion suppression: novel methodology, challenges, and opportunities. *Iran. J. Sci. Technol. Trans. Civil Eng.* **48**, 1143–1149 (2024).

[19] Harran, R., Terzis, D. & Laloui, L. Mechanics, modeling, and upscaling of biocemented soils: a review of breakthroughs and challenges. *Int. J. Geomech.* **23**, 03123004 (2023).

[20] Mitchell, J. K. & Santamarina, J. C. Biological considerations in geotechnical engineering. *J. Geotech. GeoEnviron. Eng.* **131**, 1222–1233 (2005).

[21] Wang, X. & Nackenhorst, U. A coupled bio-chemo-hydraulic model to predict porosity and permeability reduction during microbially induced calcite precipitation. *Adv. Water Resour.* **140**, 103563 (2020).

[22] Ma, G. et al. Pore-scale investigation of MICP in simplified pore structures through microfluidic tests. *Water Resour. Res.* **61**, e2024WR037807 (2025).

[23] Mobarezi, M., Nikooee, E., Owji, R. & Habibagahi, G. Enzyme-Induced carbonate precipitation as a novel remedy for expansive soils: assessing microfabric and swelling characteristics. *Geotech. Geol. Eng.* **42**, 6457–6475 (2024).

[24] Hemayati, M., Nematollahi, A., Nikooee, E., Habibagahi, G. & Niazi, A. Non-ureolytic microbially induced carbonate precipitation: Investigating a cleaner biogeotechnical engineering pathway for soil mechanical improvement. *J. Eng.* e12350 (2024). (2024).

[25] Khodadadi Tirkolaei, H., Javadi, N., Krishnan, V. & Hamdan, N. Kavazanjian jr, E. Crude urease extract for biocementation. *J. Mater. Civ. Eng.* **32**, 04020374 (2020).

[26] Nemati, M. & Voordouw, G. Modification of porous media permeability, using calcium carbonate produced enzymatically in situ. *Enzym. Microb. Technol.* **33**, 635–642 (2003).

[27] Bang, S. S., Bang, S., Frutiger, S., Nehl, L. M. & Comes, B. L. Application of novel biological technique in dust suppression. (2009).

[28] Yasuhara, H., Hayashi, K. & Okamura, M. Evolution in mechanical and hydraulic properties of calcite-cemented sand mediated by biocatalyst. In: *Geo-Frontiers 2011: Adv. Geotech. Eng.* 3984–3992 (2011).

[29] Yasuhara, H., Neupane, D., Hayashi, K. & Okamura, M. Experiments and predictions of physical properties of sand cemented by enzymatically-induced carbonate precipitation. *Soils Found.* **52**, 539–549 (2012).

[30] Majumdar, S., Sarkar, M., Chowdhury, T., Chattopadhyay, B. & Mandal, S. Use of bacterial protein powder in commercial fly Ash Pozzolana cements for high performance construction materials. (2012).

[31] Kavazanjian, E. & Hamdan, N. Enzyme induced carbonate precipitation (EICP) columns for ground improvement. in *IFCEE 2015* 2252–2261 (2015).

[32] Ma, G. et al. Strength and permeability of bentonite-assisted biocemented coarse sand. *Can. Geotech. J.* **58**, 969–981 (2021).

[33] Ma, G. et al. Influence of bacterial suspension type on the strength of biocemented sand. *Can. Geotech. J.* **59**, 2014–2021 (2022).

[34] Hamdan, N. & Kavazanjian, E. Jr Enzyme-induced carbonate mineral precipitation for fugitive dust control. *Géotechnique* **66**, 546–555 (2016).

[35] Knorr, B. *Enzyme-induced Carbonate Precipitation for the Mitigation of Fugitive Dust* (Arizona State University, 2014).

[36] Wu, M. et al. Preparation and performance evaluation of environment-friendly biological dust suppressant. *J. Clean. Prod.* **273**, 123162 (2020).

[37] Almajed, A., Lemboye, K., Arab, M. G. & Alnuaim, A. Mitigating wind erosion of sand using biopolymer-assisted EICP technique. *Soils Found.* **60**, 356–371 (2020).

[38] Dakhane, A. et al. Crack healing in cementitious mortars using enzyme-induced carbonate precipitation: quantification based on fracture response. *J. Mater. Civ. Eng.* **30**, 04018035 (2018).

[39] Arab, M. G., Omar, M., Almajed, A., Elbaz, Y. & Ahmed, A. H. Hybrid technique to produce bio-bricks using enzyme-induced carbonate precipitation (EICP) and sodium alginate biopolymer. *Constr. Build. Mater.* **284**, 122846 (2021).

[40] Martin, K. K., Khodadadi, T. H. & Kavazanjian, E. Jr Enzyme-induced carbonate precipitation: Scale-up of bio-cemented soil columns. in *Geo-Congress 2020* 96–103 (American Society of Civil Engineers Reston, VA, (2020).

[41] Dilrukshi, R. & Kawasaki, S. Effective use of plant-derived urease in the field of geoenvironmental. *Geotech. Eng. J. Civil Environ. Eng.* **6**, 2 (2016).

[42] Wang, H. et al. Induced CaCO3 mineral formation based on enzymatical calcification for bioremediation under different pressure conditions. *J. Petrol. Sci. Eng.* **216**, 110787 (2022).

[43] Wang, H. et al. Marine steel protection based on biomineralization for sustainable development of coastal cities. *Bioresour. Technol.* **428**, 132404 (2025). 40139470 10.1016/j.biortech.2025.132404

[44] Wang, H. et al. The use of N-(N-butyl)-thiophosphoric triamide to improve the efficiency of enzyme induced carbonate precipitation at high temperature. *Acta Geotech.* **18**, 5063–5081 (2023).

[45] Krajewska, B. Urease-aided calcium carbonate mineralization for engineering applications: A review. *J. Adv. Res.* **13**, 59–67 (2018). 30094083 10.1016/j.jare.2017.10.009 PMC6077181

[46] Almajed, A. A. *Enzyme Induced Carbonate Precipitation (EICP) for Soil Improvement* (Arizona State University, 2017).

[47] Javadi, N., Khodadadi, H., Hamdan, N. & Kavazanjian, E. Jr EICP treatment of soil by using urease enzyme extracted from watermelon seeds. In: *IFCEE 2018* 115–124 (2018).

[48] Park, S. S., Choi, S. G. & Nam, I. H. Effect of plant-induced calcite precipitation on the strength of sand. *J. Mater. Civ. Eng.* **26**, 06014017 (2014).

[49] Krajewska, B. & Ureases, I. Functional, catalytic and kinetic properties: A review. *J. Mol. Catal. B: Enzymatic*. **59**, 9–21 (2009).

[50] Howell, S. F. & Sumner, J. B. The specific effects of buffers upon urease activity. *J. Biol. Chem.* **104**, 619–626 (1934).

[51] Hammes, F. & Verstraete*, W. Key roles of pH and calcium metabolism in microbial carbonate precipitation. *Rev. Environ. Sci. Biotechnol.* **1**, 3–7 (2002).

[52] Dey, S., Haripavan, N., Basha, S. & Babu, G. Removal of ammonia and nitrates from contaminated water by using solid waste bio-adsorbents. *Curr. Res. Chem. Biology*. **1**, 100005 (2021).

[53] Libutti, A. & Monteleone, M. Soil vs. groundwater: the quality dilemma. Managing nitrogen leaching and salinity control under irrigated agriculture in mediterranean conditions. *Agric. Water Manage.* **186**, 40–50 (2017).

[54] Li, Y. et al. Salinity-induced concomitant increases in soil ammonia volatilization and nitrous oxide emission. *Geoderma* **361**, 114053 (2020).

[55] Porter, H., Mukherjee, A., Tuladhar, R. & Dhami, N. K. Life cycle assessment of biocement: an emerging sustainable solution? *Sustainability* **13**, 13878 (2021).

[56] Hemayati, M., Nikooee, E., Habibagahi, G., Niazi, A. & Afzali, S. F. New non-ureolytic heterotrophic microbial induced carbonate precipitation for suppression of sand Dune wind erosion. *Sci. Rep.* **13**, 5845 (2023). 37037897 10.1038/s41598-023-33070-w PMC10086056

[57] Alissandratos, A. & Easton, C. J. Biocatalysis for the application of CO2 as a chemical feedstock. *Beilstein J. Org. Chem.* **11**, 2370–2387 (2015). 26734087 10.3762/bjoc.11.259 PMC4685893

[58] Roger, M., Reed, T. C. & Sargent, F. Harnessing Escherichia coli for bio-based production of formate under pressurized H2 and CO2 gases. *Appl. Environ. Microbiol.* **87**, e00299–e00221 (2021). 34647819 10.1128/AEM.00299-21 PMC8516059

[59] Tishkov, V. I. et al. Pilot scale production and isolation of recombinant NAD+-and NADP+‐specific formate dehydrogenases. *Biotechnol. Bioeng.* **64**, 187–193 (1999). 10397854

[60] Weuster-Botz, D. et al. Continuous computer controlled production of formate dehydrogenase (FDH) and isolation on a pilot scale. *Chem. Eng. Technology: Industrial Chem.-Plant Equipment-Process Eng.-Biotechnol.*. **17**, 131–137 (1994).

[61] Katsyuba, E. & Auwerx, J. Modulating NAD + metabolism, from bench to bedside. *EMBO J.* **36**, 2670–2683 (2017). 28784597 10.15252/embj.201797135 PMC5599801

[62] McReynolds, M. R., Chellappa, K. & Baur, J. A. Age-related NAD + decline. *Exp. Gerontol.* **134**, 110888 (2020). 32097708 10.1016/j.exger.2020.110888 PMC7442590

[63] Labrou, N. E., Rigden, D. J. & Clonis, Y. D. Characterization of the NAD + binding site of Candida boidinii formate dehydrogenase by affinity labelling and site-directed mutagenesis. *Eur. J. Biochem.* **267**, 6657–6664 (2000). 11054119 10.1046/j.1432-1327.2000.01761.x

[64] Slusarczyk, H., Felber, S., Kula, M. R. & Pohl, M. Stabilization of NAD-dependent formate dehydrogenase from Candida boidinii by site‐directed mutagenesis of cysteine residues. *Eur. J. Biochem.* **267**, 1280–1289 (2000). 10691964 10.1046/j.1432-1327.2000.01123.x

[65] Yoshimoto, M., Yamasaki, R., Nakao, M. & Yamashita, T. Stabilization of formate dehydrogenase from Candida boidinii through liposome-assisted complexation with cofactors. *Enzym. Microb. Technol.* **46**, 588–593 (2010).

[66] Tishkov, V., Uglanova, S., Fedorchuk, V. & Savin, S. Influence of ion strength and pH on thermal stability of yeast formate dehydrogenase. *Acta Naturae (англоязычная версия)*. **2**, 82–87 (2010). PMC3347553 22649645

[67] Boldt, A. & Ansorge-Schumacher, M. B. Formate dehydrogenase from Rhodococcus jostii (RjFDH)–A high‐performance tool for NADH regeneration. *Adv. Synth. Catal.* **362**, 4109–4118 (2020).

[68] Nanba, H., Takaoka, Y. & Hasegawa, J. Purification and characterization of formate dehydrogenase from ancylobacter aquaticus strain KNK607M, and cloning of the gene. *Biosci. Biotechnol. Biochem.* **67**, 720–728 (2003). 12784610 10.1271/bbb.67.720

[69] Shahbazi, K., Romić, M., Ferguson, R. & Suvannang, N. Standard operating procedure for soil calcium carbonate equivalent volumetric calcimeter method. *Food Agric. Organ. United Nations Rome*, 1–13 (2020).

[70] Xue, Y., Arulrajah, A., Chu, J. & Horpibulsuk, S. Soybean urease-based EICP stabilization of washed recycled sands derived from demolition wastes cured at low temperatures. *Constr. Build. Mater.* **434**, 136735 (2024).

[71] Almajed, A., Khodadadi Tirkolaei, H. & Kavazanjian, E. Jr Baseline investigation on enzyme-induced calcium carbonate precipitation. *J. Geotech. GeoEnviron. Eng.* **144**, 04018081 (2018).

[72] Ahenkorah, I., Rahman, M. M., Karim, M. R. & Teasdale, P. R. Optimization of enzyme induced carbonate precipitation (EICP) as a ground improvement technique. In:* Geo-Congress* 552–561 (American Society of Civil Engineers Reston, VA, (2020).

[73] Miftah, A., Khodadadi Tirkolaei, H. & Bilsel, H. Biocementation of calcareous beach sand using enzymatic calcium carbonate precipitation. *Crystals* **10**, 888 (2020).

[74] Hamdan, N. M. Arizona State University Tempe, AZ, USA (2015).

[75] Almajed, A., Tirkolaei, H. K., Kavazanjian Jr, E. & Hamdan, N. Enzyme induced biocementated sand with high strength at low carbonate content. *Sci. Rep.* **9**, 1135 (2019). 30718723 10.1038/s41598-018-38361-1 PMC6362242

[76] Arab, M. G. et al. State-of-the-art review of enzyme-induced calcite precipitation (EICP) for ground improvement: applications and prospects. *Geosciences* **11**, 492 (2021).

[77] Ahenkorah, I., Rahman, M. M., Karim, M. R., Beecham, S. & Saint, C. A review of enzyme induced carbonate precipitation (EICP): the role of enzyme kinetics. *Sustainable Chem.* **2**, 92–114 (2021).

[78] Terzis, D., Hicher, P. & Laloui, L. Direct currents stimulate carbonate mineralization for soil improvement under various chemical conditions. *Sci. Rep.* **10**, 17014 (2020). 33046811 10.1038/s41598-020-73926-z PMC7552400

[79] Zhou, G. T., Jimmy, C. Y., Wang, X. C. & Zhang, L. Z. Sonochemical synthesis of aragonite-type calcium carbonate with different morphologies. *New J. Chem.* **28**, 1027–1031 (2004).

[80] Wang, X., Silbermann, C. B., Nagel, T. & Nackenhorst, U. Modelling of the elastoplastic behaviour of the bio-cemented soils using an extended modified cam clay model. *J. Rock Mech. Geotech. Eng.* **16**, 2184–2197 (2024).

[81] Mwandira, W. et al. Concurrent carbon capture and biocementation through the carbonic anhydrase (CA) activity of microorganisms-a review and outlook. *Environ. Processes*. **10**, 56 (2023).

[82] Müller, W. E. et al. Nonenzymatic transformation of amorphous CaCO3 into calcium phosphate mineral after exposure to sodium phosphate in vitro: implications for in vivo hydroxyapatite bone formation. *ChemBioChem* **16**, 1323–1332 (2015). 25871446 10.1002/cbic.201500057

[83] Wang, Y. et al. State-of-the-art review of soil erosion control by MICP and EICP techniques: problems, applications, and prospects. *Sci. Total Environ.* **912**, 169016 (2024). 38043825 10.1016/j.scitotenv.2023.169016

[84] Almajed, A. et al. Enzyme-Induced carbonate precipitation (EICP)-Based methods for ecofriendly stabilization of different types of natural sands. *J. Clean. Prod.* **274**, 122627 (2020).

[85] Alotaibi, E., Arab, M. G., Abdallah, M., Nassif, N. & Omar, M. Life cycle assessment of biocemented sands using enzyme induced carbonate precipitation (EICP) for soil stabilization applications. *Sci. Rep.* **12**, 6032 (2022). 35411057 10.1038/s41598-022-09723-7 PMC9001663

[86] Almajed, A., Khodadadi, H. & Kavazanjian, E. Jr Sisal fiber reinforcement of EICP-treated soil. in *IFCEE 2018* 29–36 (2018).

[87] Carmona, J. P., Oliveira, P. J. V. & Lemos, L. J. Biostabilization of a sandy soil using enzymatic calcium carbonate precipitation. *Procedia Eng.* **143**, 1301–1308 (2016).

[88] Oliveira, P. J. V., Freitas, L. D. & Carmona, J. P. Effect of soil type on the enzymatic calcium carbonate precipitation process used for soil improvement. *J. Mater. Civ. Eng.* **29**, 04016263 (2017).

[89] Rohy, H. et al. One phase soil bio-cementation with EICP-soil mixing. In: *Proceedings of the 4th World Congress on Civil, Structural, and Environmental Engineering (CSEE’19), Rome, Italy* 7–9 (2019).

[90] RAN, D., Watanabe, J. & Kawasaki, S. Sand cementation test using plant-derived urease and calcium phosphate compound. *Mater. Trans.* **56**, 1565–1572 (2015).

[91] Alarifi, S. A. et al. A review of enzyme-induced calcium carbonate precipitation applicability in the oil and gas industry. *Front. Bioeng. Biotechnol.* **10**, 900881 (2022). 35795168 10.3389/fbioe.2022.900881 PMC9251129

