# Supplementary material for: Non-ureolytic EICP as a novel enzymatic pathway for sustainable soil stabilization
Source: Sci Rep. 2025 Aug 1;15:28150. doi: 10.1038/s41598-025-13525-y (PMC12316990; doi:10.1038/s41598-025-13525-y)
Supplement: Supplementary file 1 — Supplementary Material 1 [file 41598_2025_13525_MOESM1_ESM.docx]

**Appendix A:**

**Effluent analysis**

To investigate the chemical composition of effluent (and its recycling potential for future studies), the effluent samples were collected in sterile barrels. The effluent was analyzed using FTIR to determine its compositional properties, and the SEM imaging and EDX analysis of the particles carried by the effluent were included to enhance the understanding of the results. The FTIR results of the effluent after the first and fifth cycles of treatment are shown in Figure A.1.

| 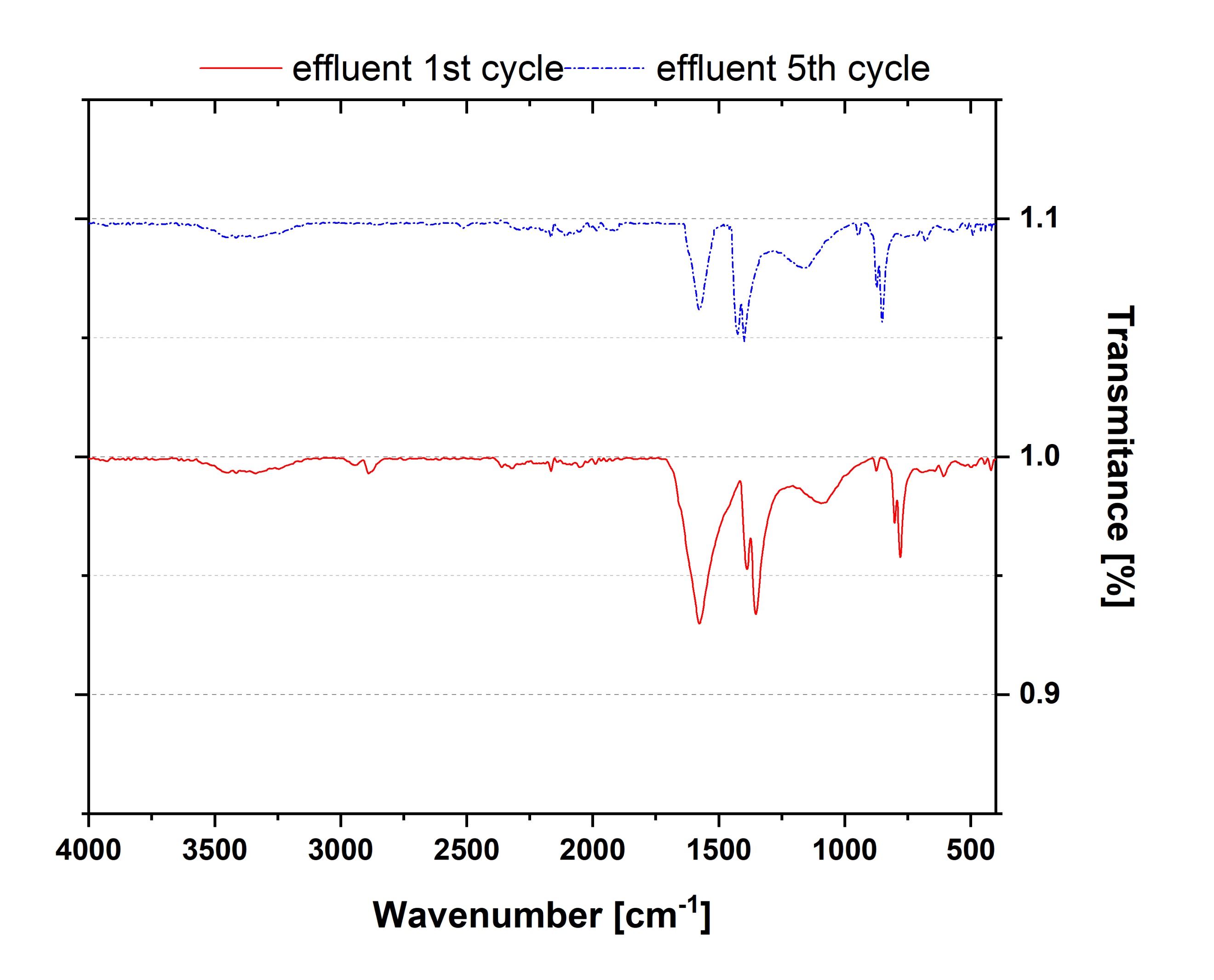 |
| --- |
| Figure A.1. The FTIR result of effluent after (a) the first cycle of treatment and (b) the fifth cycle of treatment |

As shown in Figure A.1, the FTIR peaks at 1352, 1388, and 1576 cm⁻¹ serve as valuable indicators of residual carbonate (CO₃²⁻ / HCO₃⁻) availability. In the first cycle, the FTIR peaks are sharp and visible, resulting in yields comparable to CaCO₃ precipitation. By increasing the cycles to 5, it may yield comparable CaCO₃ precipitation. As it is seen, Effluent accumulated byproducts or salts (e.g., formate if using Ca-formate). By increasing the cycles, the decrease in 1350–1570 cm⁻¹ peaks (CO₃²⁻ / HCO₃⁻) is shown ion depletion, highlighting the need for tailored reactivation strategies. The SEM and EDX results of the particles carried by effluent after the first and fifth cycles of treatment are presented in Figures A.2 and A.3.

| 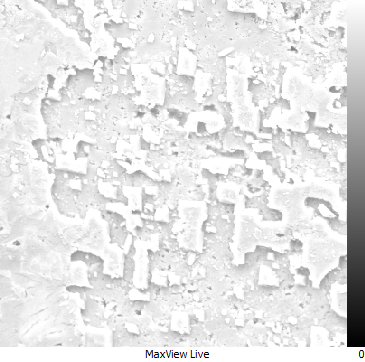 | 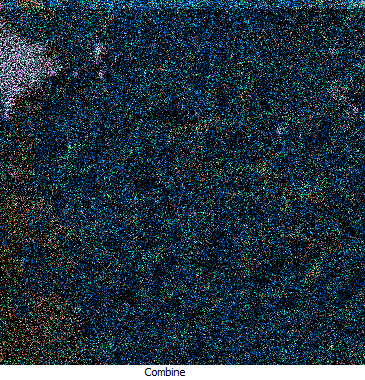 | 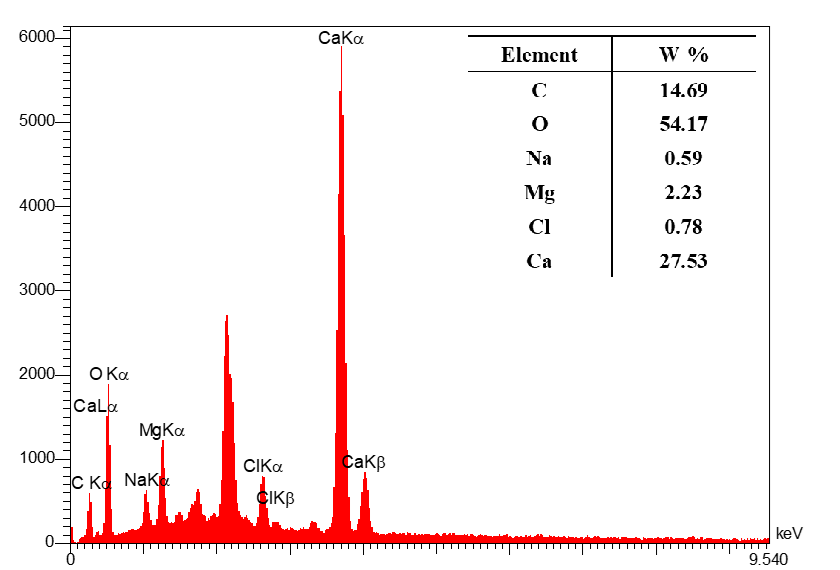 |
| --- | --- | --- |
| Figure A.2. The results of EDX analysis of the particles carried by the effluent after 1^st^ cycle of treatment, yellow region confirming the presence of calcium (Ca). | | |

| 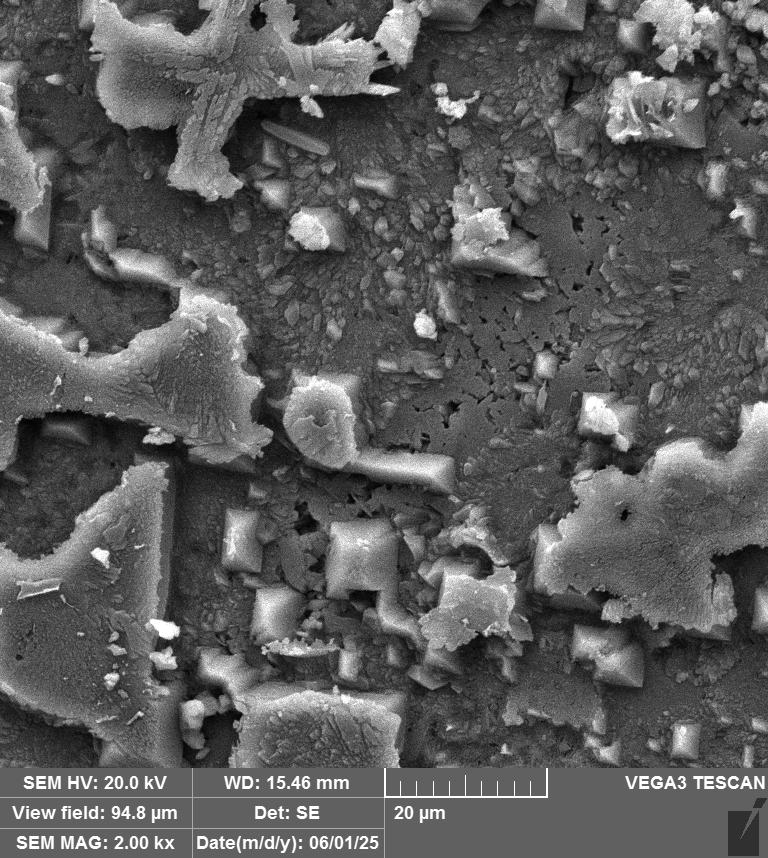 | 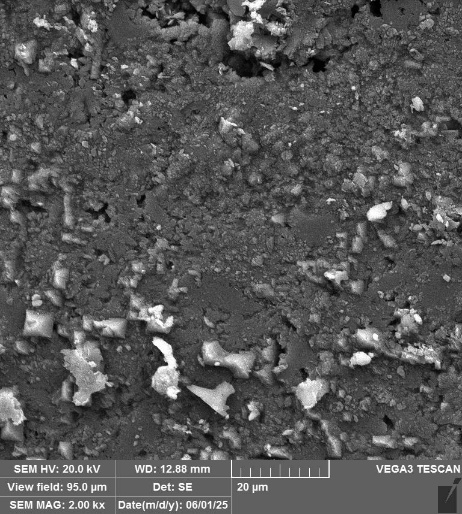 |
| --- | --- |
| (a) | (b) |
| Figure A.3. The SEM image of the particles present in the collected effluent after (a) the first cycle of treatment and (b) the fifth cycle of treatment | |

The flow rate of the output effluent was measured to indicate the effect of the precipitates on the treated soils’ permeability. Figure A.4 indicates the change in flow rate with the addition of treatment cycles. As shown, the effluent flow rate decreased from 25 to 0.05 mL/min after the fifth treatment cycle. This demonstrates a reduction in permeability due to the increase in the number of treatment cycles.

| 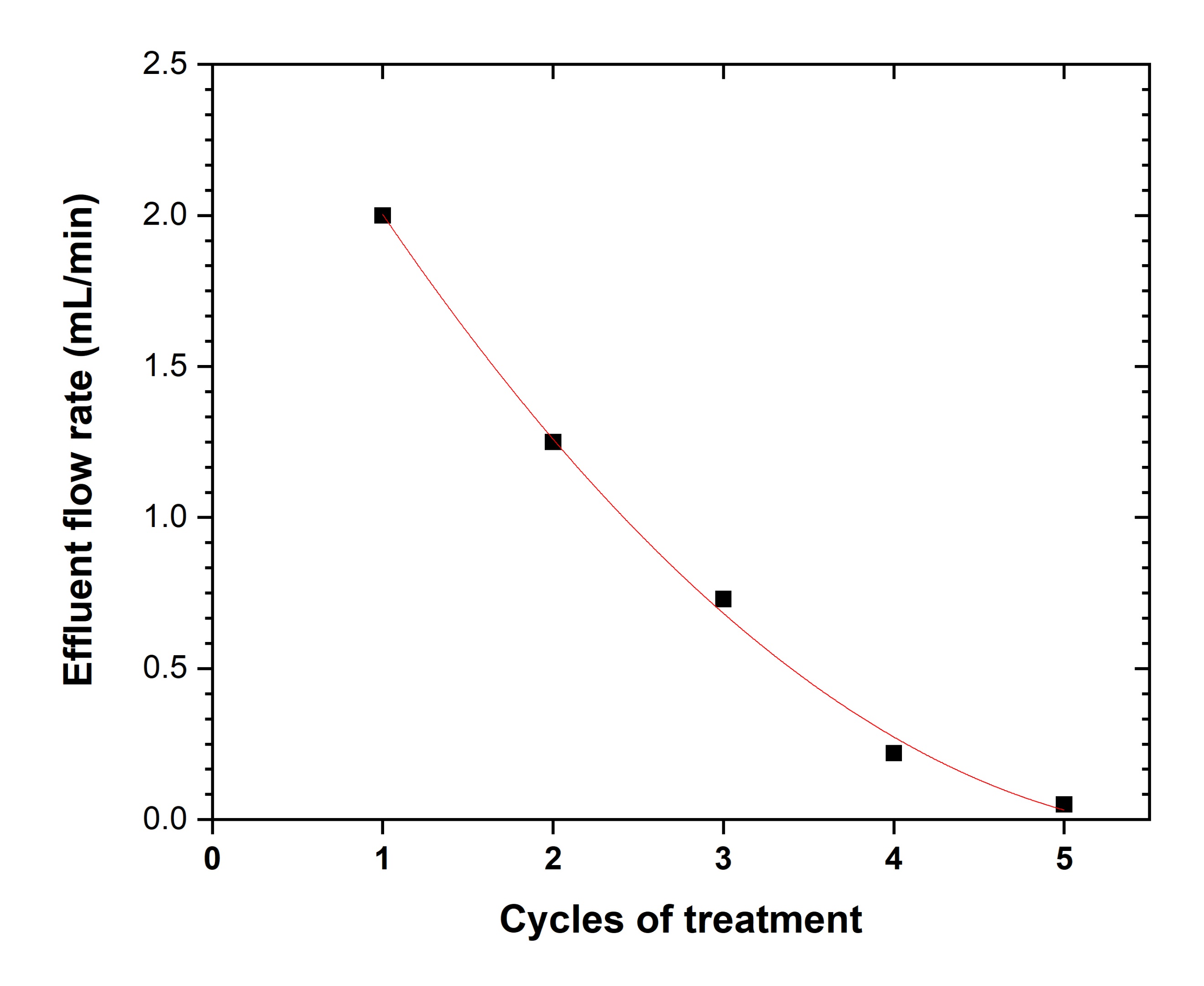 |
| --- |
| Figure A.4. The change in effluent flow rate with increasing treatment cycles |

In conclusion, it was demonstrated that the effluent from prior EICP treatment has an unconsumed calcium source from an incomplete treatment process. This effluent can be used in subsequent cycles after adding fresh enzymes. This indicates that such effluent can be reused as a source of reagents in subsequent treatment cycles.

**Appendix B:**

**Precipitation rate analysis**

To investigate the precipitation rate of the samples concerning the change in pH, the precipitation ratio is calculated over time. The precipitation ratio is defined as the relationship between the theoretical amount of calcium carbonate that can precipitate and the actual amount that precipitates in experimental conditions. It is determined by measuring the production of NADH over time in an activity test at different time intervals. The activity test process is described in section 3.1.1 of the manuscript. The change in the precipitation ratio with time is indicated in Figure B. This figure shows that the precipitation ratio at high pH concentrations increases rapidly; however, the final ratio remains below the optimal pH of 7.6.

| 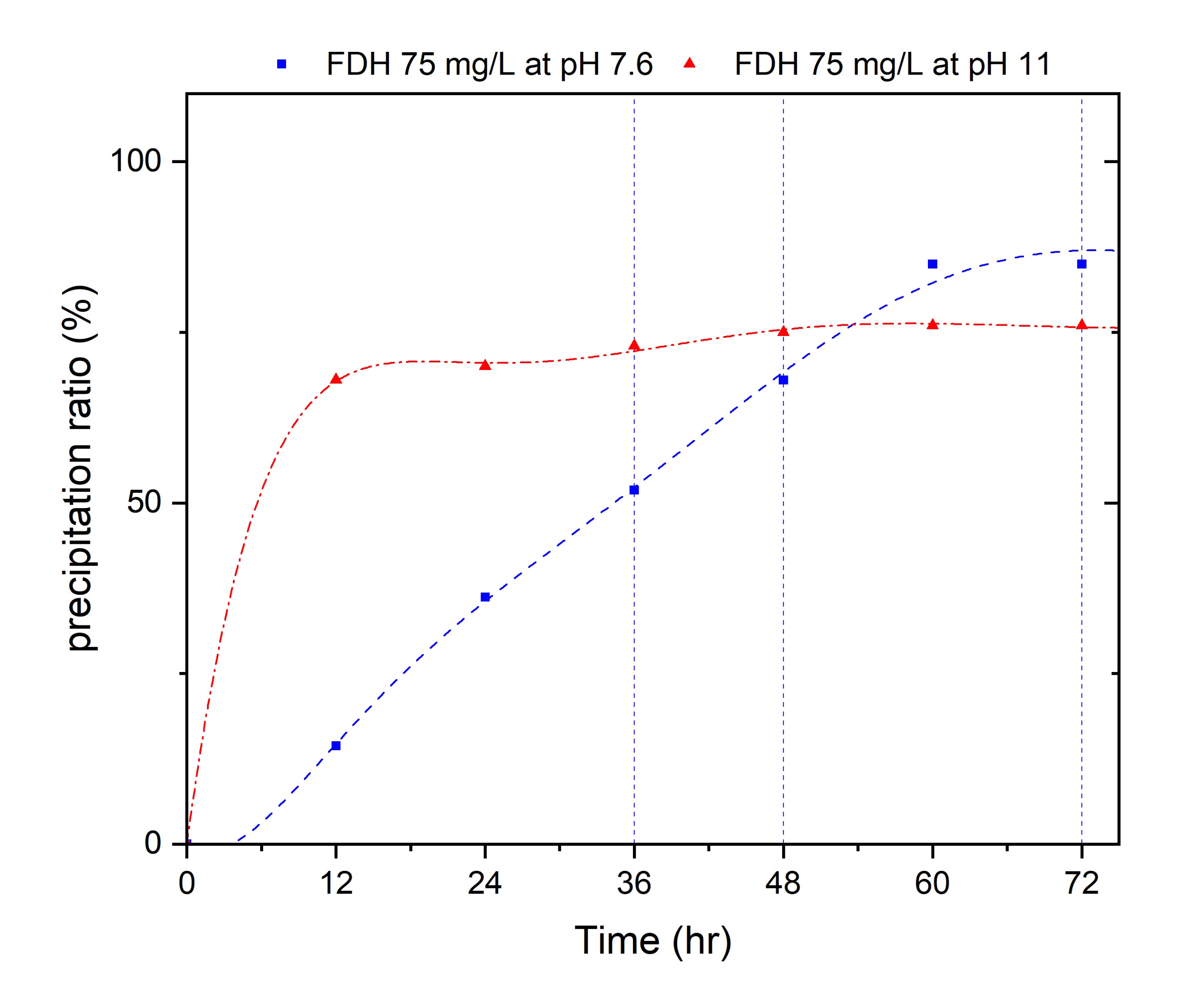 |
| --- |
| Figure B. The change in precipitation ratio with time at different pH levels |
